# Comparative Analysis of the Complete Mitochondrial Genomes of Five Species of Ricaniidae (Hemiptera: Fulgoromorpha) and Phylogenetic Implications

**DOI:** 10.3390/biology11010092

**Published:** 2022-01-07

**Authors:** Huan Zhang, Wei Fang, Xiaoyun Zhao, Xin Jiang, Adam Stroiński, Daozheng Qin

**Affiliations:** 1 Key Laboratory of Plant Protection Resources and Pest Management of the Ministry of Education, Entomological Museum, Northwest A&F University, Yangling, Xianyang 712100, China;2020060097@nwafu.edu.cn (H.Z.); F15729513368@163.com (W.F.); zhaoxiaoyun228016@163.com (X.Z.); Oasis221906@163.com (X.J.); 2 Museum and Institute of Zoology, Polish Academy of Sciences, Wilcza 64, 00-679 Warszawa, Poland; adam@miiz.waw.pl

**Keywords:** Fulgoroidea, Ricaniidae, phylogeny, mitogenomes

## Abstract

**Simple Summary:**

Although previous studies have recently explored the phylogenetic relationships among the planthopper families, the taxonomic relationships between Ricaniidae and other families of Fulgoroidea need to be further explored. Meanwhile, the morphological definitions of the two largest genera, *Pochazia* Amyot & Serville, 1843 and *Ricania* Germar, 1818 (the type genus of Ricaniidae) remain controversial, and their monophyly status has never been established. This study aims to clarify the relationship of Ricaniidae with other families of Fulgoroidea and to provide evidence to clarify the differences between these two related genera for species attribution. Our results support the monophyly of Ricaniidae and the sister group status of the two families Flatidae and Ricaniidae but fail to support the monophyly of *Pochazia* and *Ricania.* Diagnoses between these two genera cannot be resolved until more evidence is acquired. This study provides new evidence toward the phylogenetic analysis and revision of the distinguishing characteristics of related genera in this family.

**Abstract:**

Ricaniidae is a relatively small planthopper family with about 69 genera and 442 species worldwide. Members of this family occur throughout the warm temperate and tropical regions. Some species cause devastating damage to major agricultural and economic plants. However, the relationship between Ricaniidae and other families of Fulgoroidea needs to be further explored. The morphological definitions of the two biggest genera, *Pochazia* Amyot & Serville, 1843 and *Ricania* Germar, 1818 (the type genus of Ricaniidae) remain controversial. In this study, mitogenomes of five representatives in these two genera were decoded using the next-generation sequence method and genome assembly. Results showed that their complete mitogenomes are circular DNA molecules with 15,457 to 16,411 bp. All protein-coding genes (PCGs) begin with the start codon ATN, GTG or TTG and end with TAA, TAG, an incomplete stop codon single T or an incomplete stop codon single A. A lost DHU arm was discovered in the *trnS* gene of the five mitogenomes and the *trnV* gene within *Pochazia* *confusa, Pochazia guttifera* and *Ricania simulans.* The remnant tRNAs folded into clover-leaf structures. The sliding window, genetic distance, and Ka/Ks analyses indicated that the *cox1* gene is the slowest evolving and is relatively conserved. The phylogenetic tree topologies support (Delphacidae + (((Issidae + (Lophopidae + Caliscelidae)) + (Flatidae + Ricaniidae)) + (Achilidae + (Dictyopharidae + Fulgoridae)))) as the best topology, as recognized by both PhyloBayes, RAxML and MrBayes based on four data sets (PCG, PCGRNA, PCG12, PCG12RNA). The monophyly of Ricaniidae and the sister group status of two families Flatidae and Ricaniidae are supported, but all analyses failed to support the monophyly of *Pochazia* and *Ricania.* The diagnoses between these two genera cannot be resolved until more evidence is acquired.

## 1. Introduction

Ricaniidae Amyot & Audinet-Serville, 1843 is an economically important family in the superfamily Fulgoroidea. It is comprised of 442 species (3.2% of the Fulgoromorpha) with 69 genera (2.8% of the Fulgoromorpha) divided into two subfamilies [1]. This family of planthoppers is distributed throughout the warm temperate and tropical regions of the world. They are associated with both herbaceous and woody plants and have adapted to various habitats ranging from rainforests to semideserts.

Some phytophagous members of this family are major agricultural pests. For example, *Ricania speculum* (Walker, 1851), *Pochazia shantungensis* (Chou & Lu, 1977) and *Orosanga japonica* (Melichar, 1898) are major agricultural pests having a very wide range of hosts. Their damage, caused by sap suction and by egg-laying, may lead to the withering of the host tissues [2].

Research into the phylogenetic relationships of these planthopper families has been attempted in recent years, based either on morphological characters [3,4,5,6] or on molecular data [7,8,9]. However, their taxonomic relationships remain unclear.

For Ricaniidae, it has been clustered in one clade with Eurybrachidae and Lophopidae [3,4,5], or with Flatidae [6,9], or with Eurybrachidae [7], or with Caliscelidae [8]. The relationships between Ricaniidae and other families of Fulgoroidea need to be further explored.

The genera *Ricania* Germar, 1818 and *Pochazia* Amyot & Audinet-Serville, 1843 have the most abundant species (*Ricania* 82 spp., *Pochazia* 44 spp.) within the family Ricaniidae. The morphological definitions of both genera are unclear and based on external characters, mainly in the size of the forewing, the apical angle, and the ratio of apical margin and claval suture. In *Pochazia* the forewing is large, the apical angle is prominent, and the apical margin is longer than the claval suture. In *Ricania*, the forewing is relatively small, the apical angle broadly rounded, and the apical margin is nearly as long as the claval suture [10,11,12].

For example, the assignment of *Ricania shantungensis* Chou & Lu, 1977 has been controversial in recent years. It was transferred to *Pochazia* by Rahman et al. (2012) [12], but this treatment was rejected by Kwon et al. (2017) [13], Baek et al. (2020) [14] and Park and Jung (2020) [15].

Both genera (in recent definition and composition) are non-monophyletic groups (Stroiński, per. com.). Further research into the scope and definition of these taxa is needed.

Up to now, only three Ricaniid species mitogenomes (*Pochazia shantungensis*, *Ricania speculum* and *Ricania marginalis* (Walker, 1851)) have been sequenced [16,17,18]. In this study, the mitochondrial genomes of five Ricaniid species were sequenced and assembled (*Pochazia confusa* Distant, 1906; *Pochazia discreta* Melichar, 1898; *Pochazia guttifera* Walker, 1851; *Ricania simulans* (Walker, 1851) and *Ricania fumosa* (Walker, 1851)). This study aims to clarify the relationship of Ricaniidae with other families of Fulgoroidea and also provide evidence to clarify the differences between these two related genera for species attribution.

## 2. Materials and Methods

### 2.1. Sample Preparation and DNA Extraction

**Figure 1:**
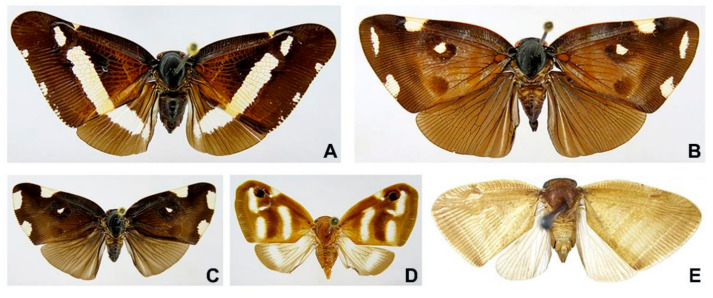
Photo plate of five Ricaniidae specimens. (**A**) *Pochazia confusa*, (**B**) *Pochazia discreta*, (**C**) *Pochazia guttifera*, (**D**) *Ricania simulans*, (**E**) *Ricania fumosa*.

Adult specimens of five Ricaniid species were studied. *Pochazia confusa* (Figure 1A), *Pochazia discreta* (Figure 1B), *Pochazia guttifera* (Figure 1C), and *Ricania fumosa* (Figure 1E) were collected in Guangdong Province (Table S1). *Ricania simulans* (Figure 1D) was collected in Hunan Province (Table S1). All specimens were preserved in 100% ethanol at −20 °C to allow DNA extraction. All specimens were identified by the first author before DNA extraction. The genomic DNA was extracted using the DNeasy DNA Extraction Kit (Qiagen).

### 2.2. Sequencing, Assembly, Annotation, and Analysis

The whole genomic DNA for each of the five Ricaniid species was sequenced once by the next-generation sequence method on the Illumina NovaSeq platform. Results were aligned with Sanger sequencing results to ensure accuracy. The quality-trimming and assembly of the paired reads were checked by Geneious v 11.0.2 with default parameters [19], employing the closely related *Ricania speculum* (Hemiptera: Ricaniidae; MT834932) [17] as a reference sequence.

The annotation of genomic features was conducted using Geneious v 11.0.2, with *Ricania speculum* and *Pochazia shantungensis* as references. The open reading frames (ORFs) Finder was created based on the invertebrate mitochondrial genetic codes. The mitogenomic maps of these five species were visualized using the CGview Server [20]. The secondary structures of tRNAs were predicted by the MITOS Web Server [21]. According to the predicted results, the tRNAs of the five species were edited using Adobe Illustrator CS2020.

PhyloSuite v 1.2.2 calculated base composition and RSCU (relative synonymous codon usage) [22]. Tandem Repeats Finder Online server was employed to obtain the tandem repeats in the control region [23]. The sliding window analysis was performed with DnaSP v 6.0 based on concatenated alignments of PCGs and rRNA genes among eight Ricaniidae mitogenomes [24]. The average non-synonymous (Ka)/synonymous (Ks) substitution rates and average genetic distances were estimated via DnaSP v 6.0 and MEGA-X based on each PCG of the eight Ricaniidae mitogenomes, respectively [25]. The mitogenomes of *Pochazia confusa*, *Pochazia discreta*, *Pochazia guttifera*, *Ricania simulans* and *Ricania fumosa* were uploaded to GenBank with accession numbers MZ617458, MZ673797, MZ617457, MZ617459, and MZ617460, respectively (Table 1).

### 2.3. Mitogenome Sequence Alignment and Analyses of Sequence Heterogeneity

Five newly sequenced Ricaniidae mitogenomes and a total of 44 known mitogenome sequences of Fulgoroidea were selected as ingroups, including 3 species of Ricaniidae, 10 species of Delphacidae, 5 species of Achilidae, 1 species of Dictyopharidae, 14 species of Fulgoridae, 2 species of Issidae, 1 species of Lophopidae, 4 species of Caliscelidae, and 4 species of Flatidae. *Populicerus populi* (Linnaeus) (Cicadellidae), *Empoascanara gracilis* Dworakowska, 1992 (Cicadellidae), *Stictocephala bisonia* Kopp & Yonke, 1977 (Membracidae), and *Tricentrus brunneus* Funkhouser, 1918 (Membracidae) were selected as outgroups (Table 1).

The extraction of 13 PCGs, 2 rRNAs, and amino acid (PCG-AA) was implemented by PhyloSuite v 1.2.2 [22]. All 13 PCGs were aligned with the G-INS-i algorithm and codon alignment mode in MAFFT 7 [26]. Alignments of two rRNAs were produced with the Q-INS-i algorithm in the MAFFT 7. Alignments of PCG-AA were produced using the G-INS-i algorithm in the MAFFT 7. We used Gblocks v 0.91b [27] to remove all gaps and poorly matched aligned sites of 13 PCGs, 2 rRNAs, and PCG-AA. The concatenated data of all alignments were performed by PhyloSuite v 1.2.2. Five various data sets were generated to reconstruct the phylogeny: (1) PCG matrix (all codon positions of PCGs), (2) PCGRNA matrix (all codon positions of PCGs and 2 rRNAs), (3) PCG12 matrix (removal of third codon position of PCGs), (4) PCG12RNA matrix (removal of third codon position of PCGs and 2 rRNAs) and (5) PCG-AA matrix (amino acid sequences of PCGs).

The sequence divergence heterogeneity of five data sets was assessed using AliGROOVE [28] with the default sliding window size. The gaps in the nucleotide data set were treated as ambiguity, and a BLOSUM62 matrix was used for a default amino acid substitution matrix.

### 2.4. Phylogenetic Analyses

The phylogenetic analyses under site-homogeneous models were reconstructed by Bayesian Inference (BI) and Maximum Likelihood (ML) methods. The optimal partitions and best models for both ML and BI trees were selected by PartitionFinder 2.1.1 (Tables S9 and S10) [29], with greedy algorithm and BIC criterion. ML analyses were conducted in IQ-TREE 1.6.5 [30] using 1000 replicates of ultrafast bootstraps. BI analyses were performed using MrBayes 3.2.6 [31], each run for 10,000,000 generations, with sampling every 100 generations. A consensus tree was calculated from the remaining samples after burn-in of the first 25% of trees.

We used PhyloBayes MPI v1.5a on CIPRES [32] to reconstruct Bayesian inferences with a site-heterogeneous CAT+GTR model and the default parameter. Two independent chains proceeded simultaneously until the runs were converged (maxdiff was <0.1). The initial 25% of the two chains were discarded as burn-in and a consensus tree was generated from the remaining samples.

## 3. Results

### 3.1. Mitogenome Organization and Base Composition

**Figure 2:**
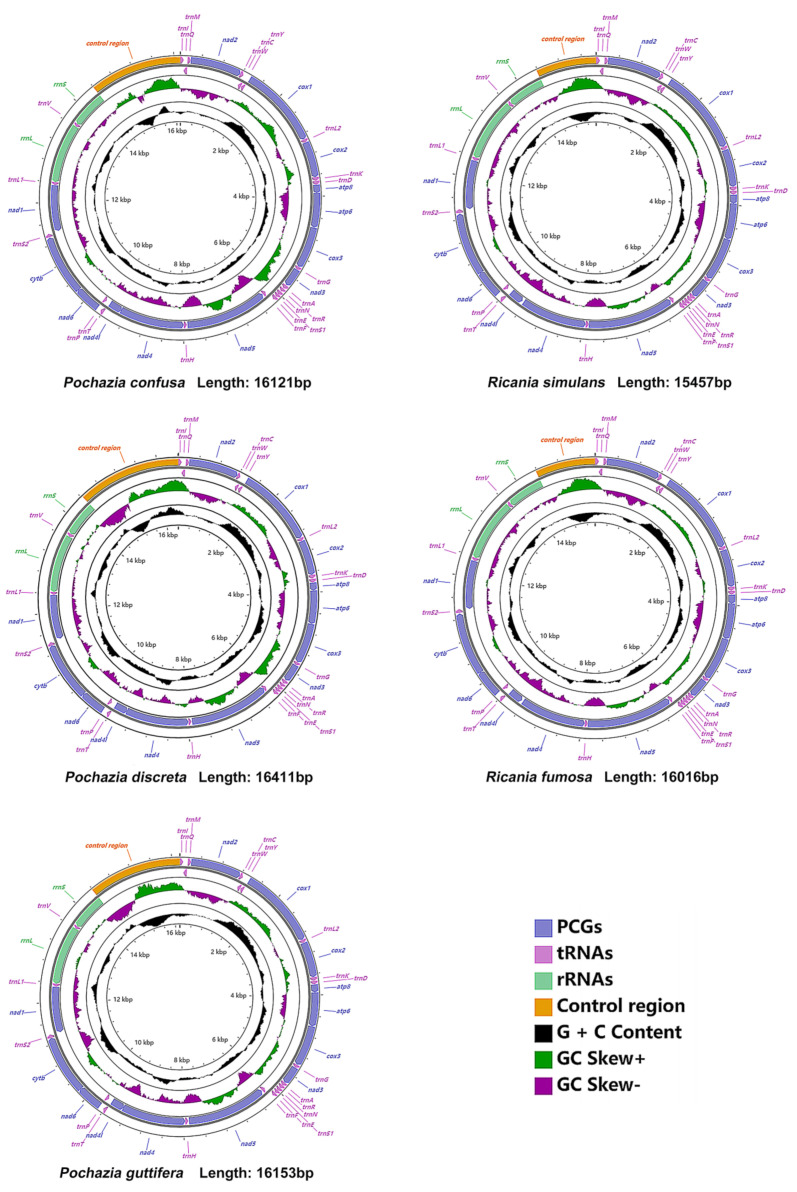
The mitochondrial genome of *Pochazia confusa, Pochazia discreta, Pochazia guttifera, Ricania simulans* and *Ricania fumosa*.

The circular complete mitogenomes of *Pochazia confusa*, *Pochazia discreta*, *Pochazia guttifera*, *Ricania simulans* and *Ricania fumosa* were 16,121, 16,411, 16,153, 15,457 and 16,016 bp in length, respectively (Figure 2). The total length of the complete mitogenomes is associated with the variation in length of the control region. The five newly sequenced mitogenomes comprised the typical 37 genes: 13 protein-coding genes (PCGs), 22 transfer RNA genes (tRNAs), two ribosomal RNA genes (rRNAs), and an A+T-rich region (control region). Gene arrangement was consistent with other planthopper mitogenomes. The majority strand (J-strand) encoded 9 PCGs and 14 tRNAs, while the remaining genes were encoded on the minority strand (N-strand) (Tables S2–S6).

The nucleotide composition (Table S7) for *Pochazia confusa* was: A = 48.4%, C = 14.3%, G = 9.0%, and T = 28.3%; for *Pochazia discreta*: A = 47.6%, C = 16.6%, G = 9.7%, and T = 26.0%; for *Pochazia guttifera*: A = 47.9%, C = 15.6%, G = 9.4%, and T = 27.2%; for *Ricania simulans*: A = 47.6%, C = 13.8%, G = 8.8%, and T = 29.7%; and for *Ricania fumosa*: A = 48.1%, C = 14.7%, G = 9.3%, and T = 28.0%.

The whole mitogenomes of five Ricaniid species presented a positive AT skew and negative GC skew. The high A+T content was observed in five Ricaniid mitogenomes with 76.7, 73.6, 75.1, 77.3, and 76.1%, respectively (Table S7). This situation has also been observed in other planthopper species.

### 3.2. Protein-Coding Genes and Codon Usage

The total length of PCGs ranged from 10,914 bp (*Pochazia confusa*) to 10,956 bp (*Ricania fumosa*) in size among these five newly sequenced Ricaniidae mitogenomes. Comparing the PCGs in the five Ricaniid mitogenomes, the A+T contents of *Pochazia confusa*, *Pochazia discreta*, *Pochazia guttifera*, *Ricania simulans,* and *Ricania fumosa* were 75.6, 72.9, 73.3, 76.4, and 75.6%, respectively. All PCGs represented a negative AT skew and GC skew. The A+T content of the third codon was highest, while that of the second codon was lowest. The AT skew and GC skew of the first codon position was highest (Table S7).

In the Ricaniid mitogenomes, most PCGs initiated with the typical start codon ATN (ATA/T/G/C), with an exception for the *nad5* gene in *Ricania simulans* and *Ricania fumosa* that began with GTG and TTG, respectively. Correspondingly, most PCGs terminated with a TAA/TAG codon, but the *cox2*, *atp6*, and *nad4* genes ended with a single T, except for *atp6* in *Ricania marginalis* using TAG as the stop codon. The *nad1* gene in *Ricania speculum*, *Pochazia shantungensis,* and *Ricania marginalis* ended respectively with a single A, single A, and TAA, whereas the five newly sequenced mitochondrial genomes terminated with T (Table S8). A large segment poly (A) appeared in *nad4* and *nad5* genes of five newly sequenced Ricaniidae mitogenomes.

**Figure 3:**
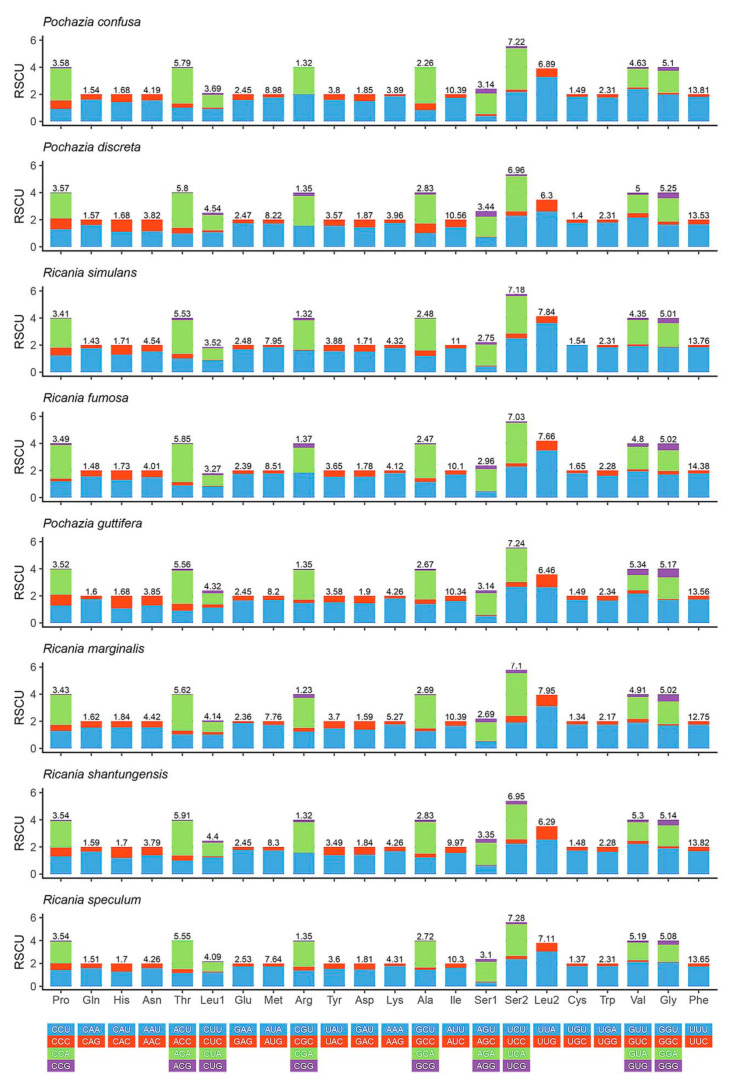
Relative synonymous codon usage (RSCU) of the mitogenomes of eight Ricaniidae species.

The RSCU (relative synonymous codon usage) of eight Ricaniidae mitogenomes is shown in Figure 3. Phe (UUU), Ile (AUU), Met (AUA), Ser (UCA), and Leu (UUA) were observed to be the most frequently used codons. The amino acid compositions were mostly A or U, indicating the strong AT bias in the whole mitochondrial genome. This codon usage pattern across these eight Ricaniidae mitogenomes was consistent with other planthoppers. However, the codon Thr (ACG) was not found in *Ricania speculum* and the codons Arg (CGG) and Ala (GCG) were not observed in *Pochazia confusa*.

### 3.3. Transfer and Ribosomal RNA Genes

The 22 tRNAs were located scattered throughout the whole mitogenome in the five newly sequenced Ricaniid species (Figure 2). Their total lengths ranged in size from 1406 bp (*Ricania simulans*) to 1424 bp (*Pochazia discreta*). The tRNAs presented a positive AT skew and GC skew in the five Ricaniid mitogenomes. These tRNAs with a heavy AT nucleotide bias reached 76.0, 74.8, 75.5, 76.1, and 76.1% in *Pochazia confusa*, *Pochazia discreta*, *Pochazia guttifera*, *Ricania simulans,* and *Ricania fumosa*, respectively (Table S7); this has also been found in other sequenced planthoppers.

The loss of the DHU arm was found in the *trnS* gene of the five Ricaniid mitogenomes and the *trnV* gene within *Pochazia confusa, Pochazia guttifera,* and *Ricania simulans.* The remnant tRNAs folded into clover-leaf structures. All five newly sequenced mitogenomes had an unpaired base in the anticodon stem of the *trnL2* and *trnR* genes. In addition, six types of unmatched base pairs, G-U, U-U, A-A, G-A, A-C, and U-C, were found in the secondary structure of tRNAs in these five Ricaniid mitogenomes. The total number of unmatched base pairs were 30 in *Pochazia confusa*, 29 in *Pochazia discreta*, 28 in *Pochazia guttifera*, 32 in *Ricania simulans* and 28 in *Ricania fumosa* (Figures S1–S5).

The total lengths of two rRNAs ranged from 1930 bp (*Pochazia confusa*) to 1941 bp (*Pochazia guttifera*) in size. The *rrnL* gene, located between *trnL1* and *trnV*, was 1206 bp in *Pochazia confusa*, 1211 bp in *Pochazia discreta*, 1217 bp in *Pochazia guttifera*, 1209 bp in *Ricania simulans,* and 1212 bp in *Ricania fumosa*. The *rrnS* gene, flanked by *trnV* and the control region, was 724 bp in *Pochazia confusa*, 722 bp in *Pochazia discreta*, 724 bp in *Pochazia guttifera*, 722 bp in *Ricania simulans,* and 722 bp in *Ricania fumosa*. The two rRNA in these five mitogenomes showed a negative AT skew and positive GC skew. In addition, *Pochazia confusa* (79.2%), *Pochazia discreta* (77.3%), *Pochazia guttifera* (77.3%), *Ricania simulans* (78.6%), and *Ricania fumosa* (77.9%) represented heavy A+T content (Tables S2–S7).

### 3.4. The Control Region

**Figure 4:**
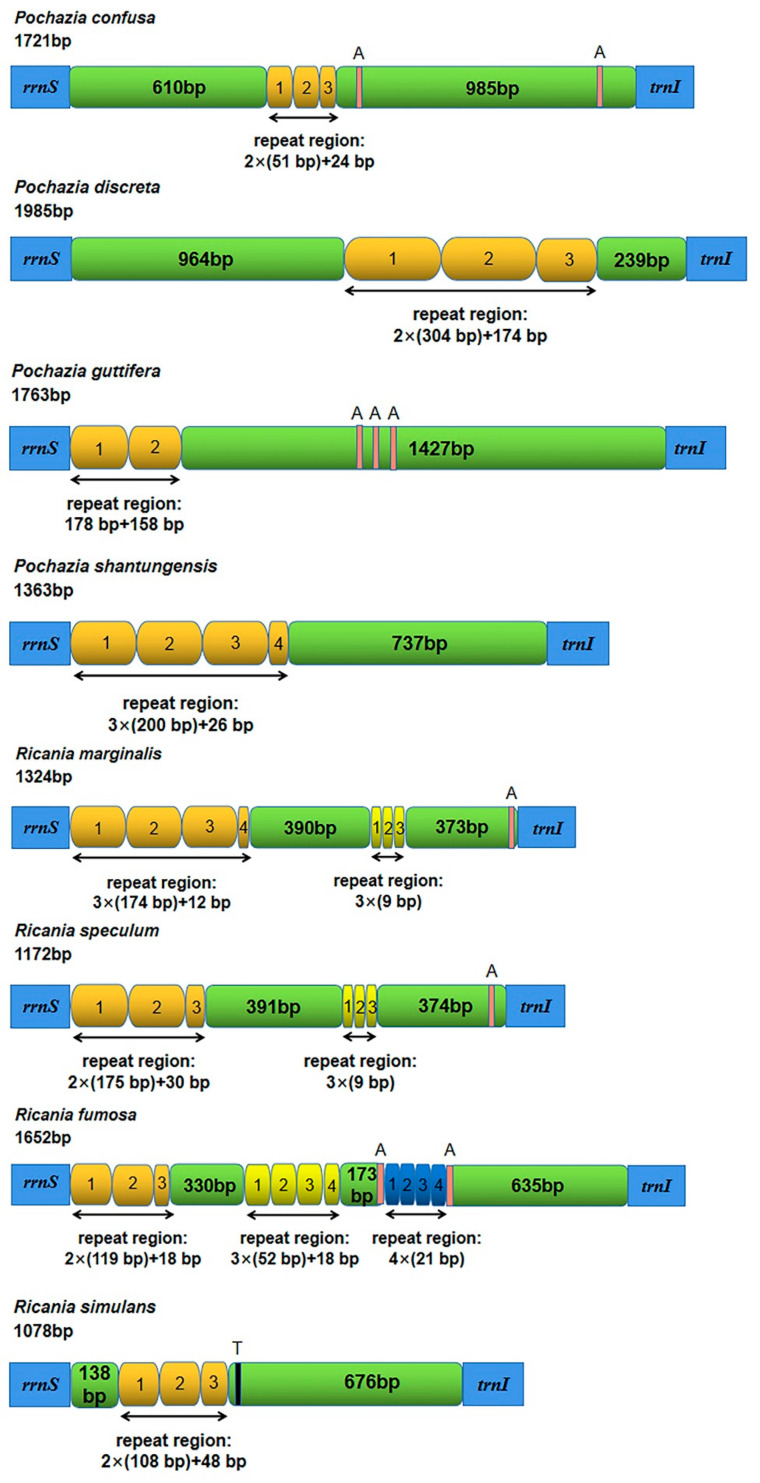
Organization of the control regions in the eight Ricaniidae mitogenomes. The chrome yellow, yellow ochre, and blue rounded rectangles indicate the tandem repeats. Non-repeat regions are represented by a green rounded rectangle. The red and black blocks are the structures of poly (A) or poly (T).

The control region, encoded on the J-strand, was located between *rrnS* and *trnI* (Figure 2). The control region of all five newly sequenced mitogenomes represented a positive AT skew and negative GC skew, except *Ricania simulans,* which presented a negative AT skew and positive GC skew. The total length of this region was 1721 bp in *Pochazia confusa*, 1985 bp in *Pochazia discreta*, 1763 bp in *Pochazia guttifera*, 1078 bp in *Ricania simulans,* and 1652 bp in *Ricania fumosa* (Tables S2–S7). Comparing tandem repeat regions of eight Ricaniid mitogenomes, the results showed that one repeat region was detected in *Pochazia confusa, Pochazia discreta*, *Pochazia guttifera, Pochazia shantungensis,* and *Ricania simulans,* and three repeat regions were present in *Ricania fumosa.* In addition, the control region of *Ricania speculum* and *Ricania marginalis* had two tandem repeat regions with a second repeat unit of “ATAATATAT”. We also found poly (A) or poly (T) in some Ricaniid species (Figure 4).

### 3.5. Nucleotide Diversity and Genetic Distance

**Figure 5:**
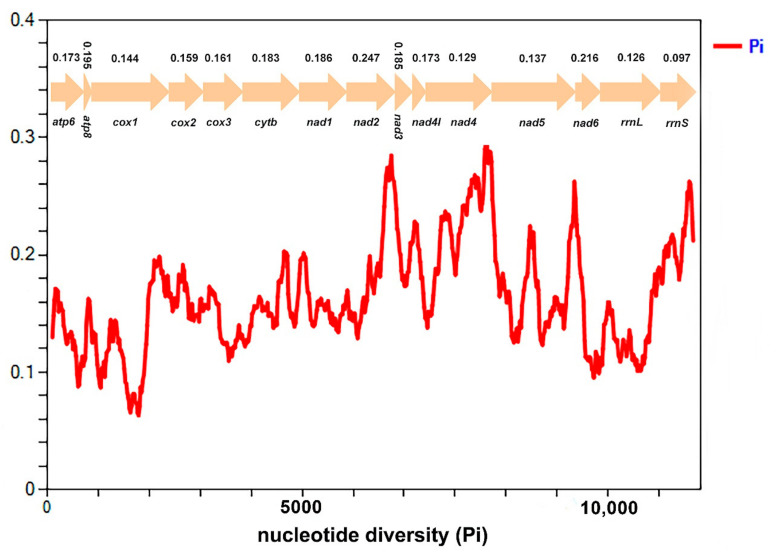
Sliding window analysis of 13 PCGs and 2 rRNAs based on eight Ricaniidae species. The red line shows the value of nucleotide diversity Pi (window size = 200 bp, step size =20 bp). The Pi value for each gene is shown in the graph.

Nucleotide diversity of 13 PCGs and 2 rRNAs is shown among eight Ricaniidae species in Figure 5. The value of nucleotide diversity ranged from 0.097 (*rrnS*) to 0.247 (*nad2*). The result indicated that *nad6* (Pi = 0.216) and *nad2* (Pi = 0.247) presented higher nucleotide diversity, whereas *nad4* (Pi = 0.129), *nad5* (Pi = 0.137), and *cox1* (Pi = 0.144) exhibited a relatively low nucleotide diversity. Two rRNAs (*rrnS* (Pi = 0.097) and *rrnL* (Pi = 0.126)) were highly conserved genes (Figure 5).

**Figure 6:**
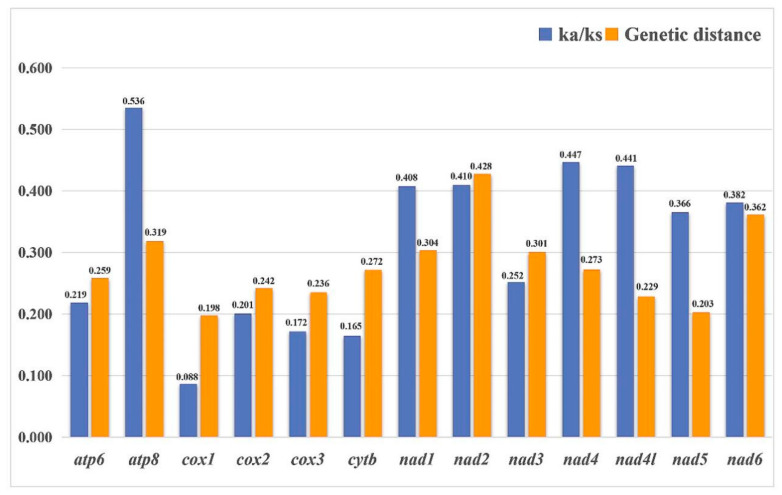
Ka/Ks rates and genetic distance (on average) of 13 PCGs were calculated among eight Ricaniidae species, using *Cromna sinensis* as a reference sequence.

The average Ka/Ks rates of 13 PCGs were calculated among eight Ricaniidae species with *Cromna sinensis* as the reference sequence. The Ka/Ks values were less than 1, representing purifying selection in each gene. The Ka/Ks ratio of *cox1* (ω = 0.088) exhibited the strongest purifying selection, while *atp8* (ω = 0.536) exhibited the weakest purifying selection. Furthermore, the average genetic distances among eight Ricaniidae species with *Cromna sinensis* as the reference sequence showed that *nad2* (0.428) was evolving comparatively fast, while *cox1* (0.198) was relatively slower (Figure 6).

**Figure 7:**
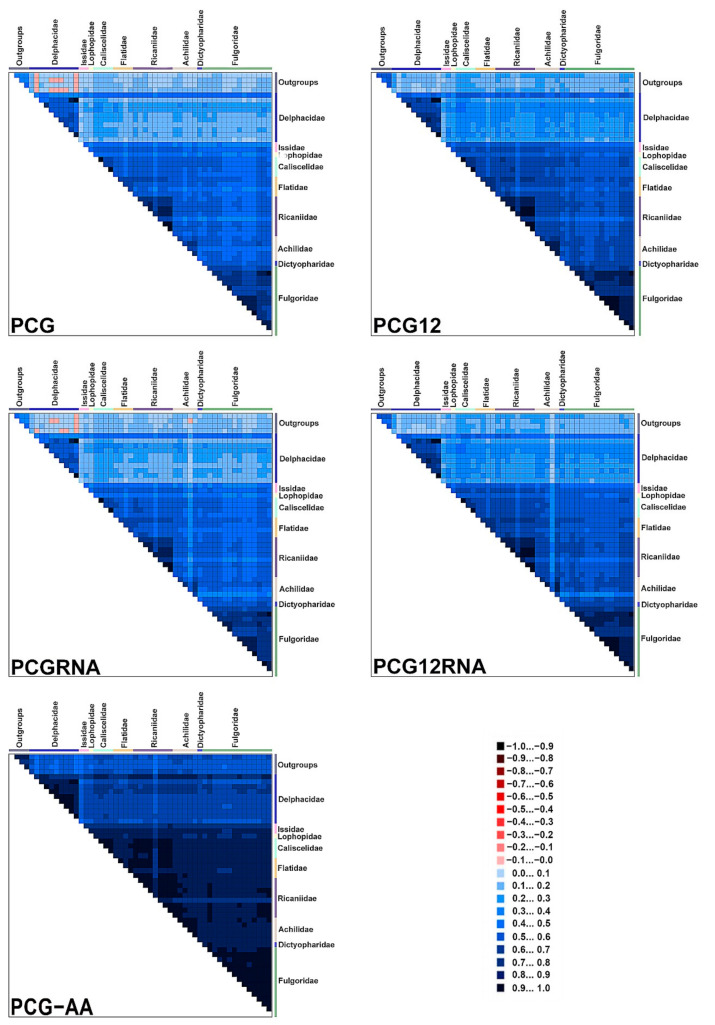
Heterogeneous sequence divergence for five different data sets with 53 taxa. Each colored square represents the pairwise Aliscore values. The scores range from −1, indicating a great difference in sequence composition (red coloring), to +1, indicating similarity to other sequence composition (blue coloring). The taxon names of different families are represented by color-coded boxes, listed on top and on the right side of the matrix.

According to this assessment, heterogeneity was higher in pairwise sequence comparisons with the Delphacidae, whereas heterogeneity was lower among other planthoppers. Results also indicated that PCG-AA had lower heterogeneity than other data sets. Comparing sequence composition heterogeneity of four data sets (PCG, PCGRNA, PCG12 and PCG12RNA), we found the third codon of PCGs had higher heterogeneity (Figure 7).

### 3.6. Phylogenetic Analyses

**Figure 8:**
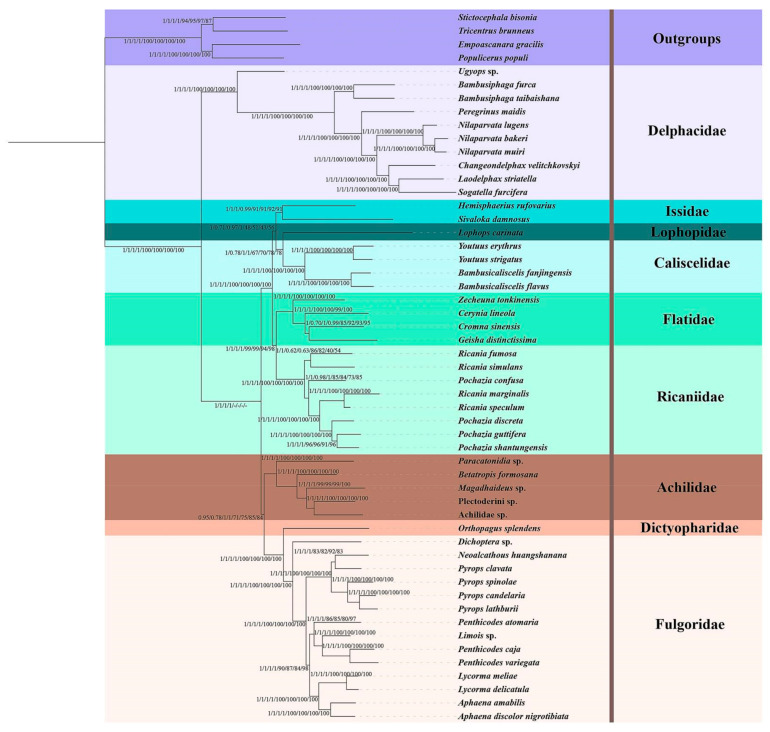
Phylogenetic tree obtained from IQ-TREE and MrBayes based on the data sets of PCG, PCGRNA, PCG12, and PCG12RNA. The numbers at nodes (from left to right) are Bayesian posterior probabilities (PPs) and then ML bootstrap support values (BS).

To establish the evolutionary relationships within Ricaniidae species, the complete mitogenomes of 44 other planthoppers species were downloaded from GenBank (Table 1). The tree topologies of the PhyloBayes, ML, and BI analyses were identical based on four data sets (PCG, PCGRNA, PCG12, PCG12RNA). These phylogenetic trees were (Delphacidae + (((Issidae + (Lophopidae + Caliscelidae)) + (Flatidae + Ricaniidae)) + (Achilidae + (Dictyopharidae + Fulgoridae)))) (Figure 8 and Figure S6). Two tree topologies based on PCG-AA were different from the above results. The tree topologies with ML and BI analyses were (Delphacidae + ((Dictyopharidae + Fulgoridae) + (Achilidae + ((Lophopidae + Caliscelidae) + (Issidae + (Flatidae + Ricaniidae)))))) (Figure S7). The tree topologies using PhyloBayes analyses were (Delphacidae + (Achilidae + ((Dictyopharidae + Fulgoridae) + (Lophopidae + ((Issidae + Caliscelidae) + (Flatidae + Ricaniidae)))))) (Figure S8). Compared to PhyloBayes, RAxML and MrBayes, a phylogeny of (Delphacidae + (((Issidae + (Lophopidae + Caliscelidae)) + (Flatidae + Ricaniidae)) + (Achilidae + (Dictyopharidae + Fulgoridae)))) was the best topology. In most results, Lophopidae and Caliscelidae were recovered as a sister lineage. All results supported the family Delphacidae as the most ancient lineage with maximum values (PP/BS = 1/100). In addition, the Ricaniidae were placed as a sister group to Flatidae with moderate nodal values in all results (BS > 94; PP > 0.78) except ML analysis of PCG-AA had a weak nodal value (BS = 55).

The relationship within Ricaniidae in all phylogenetic trees was ((*Ricania simulans* + *Ricania fumosa*) + (*Pochazia confusa* + ((*Ricania speculum* + *Ricania marginalis*) + (*Pochazia discreta* + (*Pochazia guttifera* + *Pochazia shantungensis*))))) (Figure 8 and Figures S6–S8). These results recovered *Ricania simulans* and *Ricania fumosa*, *Ricania speculum* and *Ricania marginalis* as sister taxa, respectively. Similarly, *Pochazia shantungensis* was placed as sister to *Pochazia guttifera*.

## 4. Discussion

### 4.1. Comparative Analysis of Ricaniid Mitogenomes

Current evidence shows that *Pochazia discreta* has the largest size with 16,411 bp while *Ricania simulans* has the smallest size with 15,457 bp [16,17,18]. The size variation depends on the variation of length in the control region. Here we found diversity in the repeat unit among the control region of Ricaniid species. The control region of the genus *Pochazia* had one repeat unit. There are different repeat units in the genus *Ricania*, but *Ricania speculum* and *Ricania marginalis* had two tandem repeat regions with the second repeat unit of “ATAATATAT”. Tandem repeats were also found in the control region in other species of Fulgoroidea [18,33,34,35,36,37].

This study found *cox2*, *atp6*, *nad4* ended with an incomplete T, which is universal in planthoppers [33,34,36]. In addition, *nad1* was terminated with incomplete A in *Pochazia shantungensis* and *Ricania speculum*, which rarely exists in other planthoppers. Meanwhile, a large segment poly (A), which probably makes mitochondrial genome sequence rather difficult, was found in *nad4* and *nad5* genes of all five Ricaniidae mitogenomes. It was also found in some other planthoppers species [18,33,34].

### 4.2. Nucleotide Diversity of Ricaniid Mitogenomes

When fully analyzed, the *cox1* gene can provide reliable, rapid species-level classifications and/or species identifications, which have been used by Ceotto et al., (2008), Urban and Cryan (2009), Urban et al., (2010), Gnezdilov et al., (2015), Wang et al., (2016), Huang et al., (2017), Kwon et al., (2017), and Gnezdilov et al., (2020) [13,38,39,40,41,42,43,44]. This analysis indicates that the *cox1* gene is the slowest evolving and most relatively conserved compared to other PCGs. The *nad2* gene has a relatively faster evolution rate, suggesting that the *nad2* gene would be a suitable candidate marker for species classification in Ricaniidae.

### 4.3. Phylogeny

The monophyly of Ricaniidae and the sister grouping of Ricaniidae and Flatidae were supported in all analyses. This was consistent with the explorations of Emeljanov (1990) and Song and Liang (2013) [6,9]. Within Ricaniidae, the phylogenetic relationships among eight species were stable based on different data sets and analytical methods, *Ricania speculum* was recovered as sister to *Ricania marginalis* and *Pochazia shantungensis* grouped with *Pochazia guttifera,* all with high node support. This was consistent with previous studies of Kang et al., (2020), Lee et al., (2020), Rahman et al., (2012), Bourgoin et al., (2020), and Akiner et al., (2019) [12,16,17,45,46]. In addition, all analyses here failed to support the monophyly of both *Pochazia* and *Ricania*, which was congruent with the result of Akiner et al., (2019) [46]. Therefore, the diagnoses between these two genera cannot be resolved until more evidence is acquired.

Prior to this study, two mitochondrial data sets from *Pochazia shantungensis* and *Ricania speculum* were found in GeneBank. We think the two mitochondrial sequences of *Ricania speculum* (*Ricania speculum*-KX371891 and *Ricania speculum*-MT834932) are from the same species because the similarity in the two sequences (without the control region) is 99.0%; therefore, any of the two mitochondrial sequences can be used in this analysis. The similarity in the two mitochondrial sequences of *Pochazia shantungensis* (without the control region) (*Ricania shantungensis*-MW036196 and *Ricania shantungensis*-MT898421) from Korea is 93.6%. This genetic gap seemed too big, so we compared the *cox1* gene of *Pochazia shantungensis* from China and the *cox1* gene of two mitochondrial data sets of *Pochazia shantungensis* from Korea and found that *Ricania shantungensis*-MT898421 and *Pochazia shantungensis* from China had a higher similarity ratio (97.7%) and were therefore used in this analysis.

The results presented in this paper are the beginning of a new period of research on the phylogenetic position of the family Ricaniidae and its phylogeny and evolution as well as genetic diversity within the family and between species. In addition, the species used in this study will constitute the basis for in-depth research on population and species variability for species that are listed as pests. For example, for *Pochazia shantungensis* more than 200 host plants (81 families, 157 genera) have currently been reported, and for *Ricania speculum*, more than 140 host plants (54 families, 108 genera) are known (Stroiński, unpublished). Are they related and how? Do they belong to the same genus? Further molecular studies from a larger area of distribution will answer this question.

## Figures and Tables

**Table 1:** Taxa used in this study.

Figure Taxon	Species	GenBank Number	References
Outgroups			
Cicadellidae	*Empoascanara gracilis* Dworakowska	MT576649	Unpublished
	*Populicerus populi* (Linnaeus)	MH492318	Wang et al., (2018)
Membracidae	*Stictocephala bisonia* Kopp & Yonke	MW342606	Yu et al., (2021)
	*Tricentrus brunneus* Funkhouser	NC_044708	Hu et al., (2019)
Ingroup			
Delphacidae	*Ugyops* sp.	MH352481	Yu & Liang (2018)
	*Bambusiphaga furca* Huang & Tian	NC_052689	Huang et al., (2020)
	*Bambusiphaga taibaishana* Qin	NC_052690	Huang et al., (2020)
	*Changeondelphax velitchkovskyi* (Melichar)	NC_037181	Huang & Qin (2017)
	*Peregrinus maidis* (Ashmead)	NC_037182	Huang & Qin (2017)
	*Sogatella furcifera* (Horváth)	KC512915	Zhang et al., (2014)
	*Laodelphax striatella* (Fallén)	JX880068	Zhang et al., (2013)
	*Nilaparvata bakeri* (Muir)	NC_033388	Unpublished
	*Nilaparvata lugens* (Stål)	NC_021748	Unpublished
	*Nilaparvata muiri* China	JN563998	Lv et al., (2015)
Achilidae	Achilidae sp.	MH324929	Xu et al., (2019)
	*Betatropis formosana* Matsumura	MH324927	Xu et al., (2019)
	Plectoderini sp.	MH324930	Xu et al., (2019)
	*Paracatonidia* sp.	MH324931	Xu et al., (2019)
	*Magadhaideus* sp.	MH324928	Xu et al., (2019)
Dictyopharidae	*Orthopagus splendens* (Germar)	MW441850	Unpublished
Fulgoridae	*Aphaena amabilis* (Hope)	NC_045075	Wang et al., (2019)
	*Aphaena discolor nigrotibiata* Schmidt	MN025523	Wang et al., (2019)
	*Lycorma delicatula* (White)	MN607209	Unpublished
	*Lycorma meliae* Kato	MT079725	Du et al., (2021)
	*Penthicodes atomaria* (Weber)	MW662662	Wang et al., (2021)
	*Penthicodes variegata* (Guérin-Méneville)	MW662664	Wang et al., (2021)
	*Penthicodes caja* (Walker)	MW662663	Wang et al., (2021)
	*Limois* sp.	MW662660	Wang et al., (2021)
	*Neoalcathous huangshanana* Wang & Huang	MW662661	Wang et al., (2021)
	*Dichoptera* sp.	MW662659	Wang et al., (2021)
	*Pyrops candelaria* (Linné)	MW355618	Duan & Hu (2021)
	*Pyrops clavatus* (Westwood)	MW662665	Wang et al., (2021)
	*Pyrops lathburii* (Kirby)	MW662666	Wang et al., (2021)
	*Pyrops spinolae* (Westwood)	MW662667	Wang et al., (2021)
Issidae	*Sivaloka damnosus* (Chou & Lu)	NC_014286	Song et al., (2010)
	*Hemisphaerius rufovarius* Walker	MT210096	Yang et al., (2020)
Lophopidae	*Lophops carinata* (Kirby)	NC_053739	Xu & Chen (2021)
Caliscelidae	*Bambusicaliscelis fanjingensis* Chen & Zhang	MW281859	Gong et al., (2021)
	*Bambusicaliscelis flavus* Chen & Gong	MW281858	Gong et al., (2021)
	*Youtuus erythrus* Gong, Yang & Chen	MW281861	Gong et al., (2021)
	*Youtuus strigatus* Gong, Yang & Chen	MW281860	Gong et al., (2021)
Flatidae	*Cromna sinensis* (Walker)	MW872012	Ai et al., (2021)
	*Cerynia lineola* Melichar	MW872011	Ai et al., (2021)
	*Geisha distinctissima* (Walker)	FJ230961	Song & Liang (2009)
	*Zecheuna tonkinensis* Zia	MW872013	Ai et al., (2021)
Ricaniidae	*Pochazia confusa* Distant	MZ617458	This study
	*Pochazia discreta* Melichar	MZ673797	This study
	*Pochazia guttifera* Walker	MZ617457	This study
	*Pochazia shantungensis* (Chou & Lu)	MT898421	Kang et al., (2020)
	*Ricania speculum* (Walker)	MT834932	Lee et al., (2020)
	*Ricania fumosa* (Walker)	MZ617460	This study
	*Ricania marginalis* (Walker)	NC_019597	Song et al., (2012)
	*Ricania simulans* (Walker)	MZ617459	This study

## Data Availability

Please see the section Supplementary Materials.

## Supplementary Materials

The following supporting information can be downloaded at: https://www.mdpi.com/article/10.3390/biology11010092/s1, Figure S1. The secondary structure for the tRNAs of *Pochazia confusa*. Figure S2. The secondary structure for the tRNAs of *Pochazia discreta*. Figure S3. The secondary structure for the tRNAs of *Pochazia guttifera*. Figure S4. The secondary structure for the tRNAs of *Ricania simulans*. Figure S5. The secondary structure for the tRNAs of *Ricania fumosa*. Figure S6. Phylogenetic trees obtained from PhyloBayes based on the data sets of PCG, PCGRNA, PCG12 and PCG12RNA. Figure S7. Phylogenetic trees obtained from IQ-TREE and MrBayes based on the data sets of PCG-AA. Figure S8. Phylogenetic trees obtained from PhyloBayes based on the data sets of PCG-AA. Table S1. Species investigated and their related information. Table S2. Mitogenomic organization of *Pochazia confusa*. Table S3. Mitogenomic organization of *Pochazia discreta*. Table S4. Mitogenomic organization of *Pochazia guttifera*. Table S5. Mitogenomic organization of *Ricania simulans*. Table S6. Mitogenomic organization of *Ricania fumosa*. Table S7. Nucleotide composition of mitogenomes of the five species. Table S8. Start and stop codons of eight Ricaniidae mitochondrial genomes. Table S9. Best partitioning schemes and models based on five data sets for IQ-TREE analysis. Table S10. Best partitioning schemes and models based on five data sets for MrBayes analysis.
