# Supplementary material for: Comparative Analysis of the Complete Mitochondrial Genomes of Five Species of Ricaniidae (Hemiptera: Fulgoromorpha) and Phylogenetic Implications
Source: Biology (Basel). 2022 Jan 7;11(1):92. doi: 10.3390/biology11010092 (PMC8772989; doi:10.3390/biology11010092)
Supplement: Supplementary file 1 [file biology-11-00092-s001.zip › Supplementary Tables S1-S10.pdf]

Table S1. Species investigated and their related information.

Species	Date	Distribution	Geographic coordinates
Pochazia confusa Distant	20 Jul. 2020	Chebaling National Nature Reserve, Shaoguan, Guangdong, China	24.731204 °N, 114.267436 °E
Pochazia discreta Melichar	26 Jul. 2020	Nanling Forest Park, Ruyuan, Shaoguan, Guangdong, China	24.926202 °N, 113.092558 °E
Pochazia guttifera Walker	26 Jul. 2020	Nanling Forest Park, Ruyuan, Shaoguan, Guangdong, China	24.926202 °N, 113.092558 °E
Ricania simulans (Walker)	15 Jul. 2020	Dupangling Nature Reserve, Yongzhou, Hunan, China	25.324460 °N, 111.158767 °E
Ricania fumosa (Walker)	8 Aug. 2018	Baishuizhai Scenic Area, Guangzhou, Guangdong, China	23.599255 °N, 113.771154 °E

Table S2. Mitogenomic organization of *Pochazia confusa*.

Gene	Position		Size(bp)	Codon		Strand
	From	To		Start	Stop	
trnI	1	64	64			J
trnQ	67	135	69			N
trnM	137	199	63			J
nad2	200	1165	966	ATT	TAA	J
trnW	1164	1228	65			J
trnC	1221	1283	63			N
trnY	1292	1354	63			N
cox1	1370	2905	1536	ATG	TAA	J
trnL2	2910	2972	63			J
cox2	2973	3645	673	ATA	T	J
trnK	3646	3715	70			J
trnD	3718	3788	71			J
atp8	3789	3944	156	ATA	TAA	J
atp6	3941	4589	649	ATA	T	J
cox3	4590	5372	783	ATG	TAA	J
trnG	5376	5436	61			J
nad3	5437	5784	348	ATA	TAA	J
trnA	5797	5865	69			J
trnR	5877	5937	61			J
trnN	5951	6015	65			J
trnS1	6015	6074	60			J
trnE	6080	6144	65			J
trnF	6164	6228	65			N
nad5	6243	7913	1671	ATG	TAA	N
trnH	7930	7990	61			N
nad4	7992	9312	1321	ATG	T	N
nad4L	9306	9575	270	ATG	TAA	N
trnT	9591	9653	63			J
trnP	9666	9727	62			N
nad6	9729	10223	495	ATA	TAA	J
cytb	10216	11340	1125	ATG	TAA	J
trnS2	11349	11412	64			J
nad1	11419	12343	925	ATG	T	N
trnL1	12345	12407	63			N
rrnL	12408	13613	1206			N
trnV	13614	13676	63			N
rrnS	13677	14400	724			N
CR	14401	16121	1721			J

Table S3. Mitogenomic organization of *Pochazia discreta*.

Gene	Position		Size(bp)	Codon		Strand
	From	To		Start	Stop	
trnI	1	64	64			J
trnQ	69	137	69			N
trnM	148	211	64			J
nad2	212	1177	966	ATT	TAA	J
trnW	1177	1239	63			J
trnC	1232	1294	63			N
trnY	1312	1377	66			N
cox1	1383	2918	1536	ATG	TAA	J
trnL2	2921	2983	63			J
cox2	2984	3665	682	ATA	T	J
trnK	3666	3735	70			J
trnD	3745	3814	70			J
atp8	3815	3970	156	ATT	TAA	J
atp6	3967	4615	649	ATA	T	J
cox3	4616	5398	783	ATG	TAA	J
trnG	5406	5469	64			J
nad3	5470	5817	348	ATA	TAA	J
trnA	5828	5893	66			J
trnR	5904	5964	61			J
trnN	5972	6036	65			J
trnS1	6036	6094	59			J
trnE	6097	6160	64			J
trnF	6180	6244	65			N
nad5	6239	7915	1677	ATG	TAG	N
trnH	7932	7993	62			N
nad4	7995	9318	1324	ATG	T	N
nad4L	9312	9581	270	ATG	TAA	N
trnT	9593	9656	64			J
trnP	9682	9745	64			N
nad6	9747	10241	495	ATA	TAA	J
cytb	10234	11364	1131	ATG	TAA	J
trnS2	11364	11429	66			J
nad1	11436	12360	925	ATG	T	N
trnL1	12362	12425	64			N
rrnL	12426	13636	1211			N
trnV	13637	13704	68			N
rrnS	13705	14426	722			N
CR	14427	16411	1985			J

Table S4. Mitogenomic organization of *Pochazia guttifera*.

Gene	Position		Size(bp)	Codon		Strand
	From	To		Start	Stop	
trnI	1	64	64			J
trnQ	74	142	69			N
trnM	154	217	64			J
nad2	218	1183	966	ATT	TAA	J
trnW	1183	1246	64			J
trnC	1239	1301	63			N
trnY	1312	1375	64			N
cox1	1381	2916	1536	ATG	TAA	J
trnL2	2919	2981	63			J
cox2	2982	3663	682	ATA	T	J
trnK	3664	3733	70			J
trnD	3737	3801	65			J
atp8	3802	3957	156	ATT	TAA	J
atp6	3954	4602	649	ATA	T	J
cox3	4603	5385	783	ATG	TAA	J
trnG	5391	5452	62			J
nad3	5453	5800	348	ATA	TAA	J
trnA	5803	5868	66			J
trnR	5869	5934	66			J
trnN	5937	6002	66			J
trnS1	6002	6060	59			J
trnE	6069	6131	63			J
trnF	6152	6216	65			N
nad5	6234	7886	1653	ATG	TAA	N
trnH	7903	7964	62			N
nad4	7966	9289	1324	ATG	T	N
nad4L	9283	9552	270	ATG	TAA	N
trnT	9557	9620	64			J
trnP	9640	9703	64			N
nad6	9705	10199	495	ATA	TAA	J
cytb	10192	11322	1131	ATG	TAA	J
trnS2	11325	11391	67			J
nad1	11380	12322	943	ATG	T	N
trnL1	12324	12387	64			N
rrnL	12388	13604	1217			N
trnV	13605	13666	62			N
rrnS	13667	14390	724			N
CR	14391	16153	1763			J

Table S5. Mitogenomic organization of *Ricania simulans*.

Gene	Position		Size(bp)	Codon		Strand
	From	To		Start	Stop	
trnI	1	67	67			J
trnQ	73	141	69			N
trnM	153	216	64			J
nad2	217	1188	972	ATT	TAA	J
trnW	1187	1249	63			J
trnC	1242	1304	63			N
trnY	1312	1376	65			N
cox1	1392	2927	1536	ATG	TAA	J
trnL2	2929	2991	63			J
cox2	2992	3667	676	ATA	T	J
trnK	3668	3737	70			J
trnD	3741	3809	69			J
atp8	3810	3971	162	ATA	TAA	J
atp6	3968	4616	649	ATA	T	J
cox3	4617	5399	783	ATG	TAA	J
trnG	5400	5460	61			J
nad3	5461	5808	348	ATA	TAG	J
trnA	5807	5870	64			J
trnR	5871	5931	61			J
trnN	5934	5996	63			J
trnS1	5996	6054	59			J
trnE	6054	6116	63			J
trnF	6119	6181	63			N
nad5	6190	7869	1680	GTG	TAA	N
trnH	7870	7930	61			N
nad4	7932	9222	1291	ATG	T	N
nad4L	9257	9526	270	ATT	TAA	N
trnT	9544	9605	62			J
trnP	9626	9688	63			N
nad6	9690	10184	495	ATC	TAA	J
cytb	10177	11307	1131	ATG	TAA	J
trnS2	11324	11389	66			J
nad1	11378	12320	943	ATG	T	N
trnL1	12322	12386	65			N
rrnL	12387	13595	1209			N
trnV	13596	13657	62			N
rrnS	13658	14379	722			N
CR	14380	15457	1078			J

Table S6. Mitogenomic organization of *Ricania fumosa*.

Gene	Position		Size(bp)	Codon		Strand
	From	To		Start	Stop	
trnI	1	63	63			J
trnQ	80	148	69			N
trnM	151	214	64			J
nad2	215	1183	969	ATT	TAA	J
trnW	1183	1245	63			J
trnC	1238	1300	63			N
trnY	1306	1370	65			N
cox1	1374	2909	1536	ATG	TAA	J
trnL2	2910	2972	63			J
cox2	2973	3648	676	ATA	T	J
trnK	3649	3718	70			J
trnD	3719	3788	70			J
atp8	3789	3947	159	ATT	TAA	J
atp6	3944	4592	649	ATA	T	J
cox3	4593	5375	783	ATG	TAA	J
trnG	5390	5450	61			J
nad3	5451	5798	348	ATA	TAA	J
trnA	5802	5868	67			J
trnR	5869	5928	60			J
trnN	5930	5993	64			J
trnS1	5993	6052	60			J
trnE	6055	6119	65			J
trnF	6118	6180	63			N
nad5	6189	7877	1689	TTG	TAG	N
trnH	7871	7932	62			N
nad4	7934	9254	1321	ATG	T	N
nad4L	9248	9520	273	ATG	TAA	N
trnT	9540	9601	62			J
trnP	9615	9678	64			N
nad6	9680	10168	489	ATA	TAA	J
cytb	10179	11303	1125	ATG	TAA	J
trnS2	11306	11368	63			J
nad1	11357	12299	943	ATG	T	N
trnL1	12301	12366	66			N
rrnL	12367	13578	1212			N
trnV	13579	13642	64			N
rrnS	13643	14364	722			N
CR	14365	16016	1652			J

Table S7. Nucleotide composition of mitogenomes of the five species in this study.

Regions	Size (bp)	T(U)%	C%	A%	G%	AT (%)	GC (%)	AT skew	GC skew
Pochazia confusa									
Full genome	16121	28.3	14.3	48.4	9.0	76.7	23.3	0.262	-0.224
PCGs	10914	43.0	12.8	32.6	11.6	75.6	24.4	-0.137	-0.049
1st codon position	3638	35.7	11.8	36.3	16.2	72.0	28.0	0.008	0.159
2nd codon position	3638	48.3	18.8	19.6	13.3	67.9	32.1	-0.422	-0.170
3rd codon position	3638	45.1	7.7	42.1	5.1	87.2	12.8	-0.035	-0.202
tRNAs	1413	35.2	10.3	40.8	13.6	76.0	23.9	0.073	0.136
rRNAs	1930	50.8	7.4	28.4	13.3	79.2	20.7	-0.282	0.285
Control Region	1721	32.4	10.9	47.3	9.5	79.7	20.4	0.187	-0.069
Pochazia discreta									
Full genome	16411	26.0	16.6	47.6	9.7	73.6	26.3	0.293	-0.264
PCGs	10938	41.3	14.7	31.6	12.4	72.9	27.1	-0.134	-0.084
1st codon position	3646	34.2	12.7	35.7	17.4	69.9	30.1	0.021	0.157
2nd codon position	3646	48.0	19.1	19.1	13.7	67.1	32.8	-0.430	-0.165
3rd codon position	3646	41.6	12.4	39.8	6.2	81.4	18.6	-0.022	-0.331
tRNAs	1424	34.8	10.7	40.0	14.5	74.8	25.2	0.069	0.151
rRNAs	1933	50.6	8.2	26.7	14.4	77.3	22.6	-0.308	0.274
Control Region	1985	30.0	16.5	42.8	10.7	72.8	27.2	0.175	-0.215
Pochazia guttifera									
Full genome	16153	27.2	15.6	47.9	9.4	75.1	25.0	0.276	-0.248
PCGs	10932	42.2	14.0	31.1	12.6	73.3	26.6	-0.151	-0.053
1st codon position	3644	34.8	12.4	35.3	17.5	70.1	29.9	0.006	0.169
2nd codon position	3644	48.1	18.9	19.5	13.4	67.6	32.3	-0.423	-0.169
3rd codon position	3644	43.7	10.7	38.6	6.9	82.3	17.6	-0.062	-0.215
tRNAs	1416	35.0	10.6	40.5	13.8	75.5	24.4	0.073	0.133
rRNAs	1941	50.3	8.4	27.0	14.2	77.3	22.6	-0.301	0.257
Control Region	1763	31.6	11.5	50.0	7.0	81.6	18.5	0.225	-0.243
Ricania simulans									
Full genome	15457	29.7	13.8	47.6	8.8	77.3	22.6	0.231	-0.222
PCGs	10932	43.6	12.5	32.8	11.1	76.4	23.6	-0.141	-0.060
1st codon position	3644	36.7	11.4	36.0	16.0	72.7	27.4	-0.009	0.169
2nd codon position	3644	48.3	18.6	20.3	12.9	68.6	31.5	-0.409	-0.180
3rd codon position	3644	45.9	7.5	42.3	4.3	88.2	11.8	-0.041	-0.268
tRNAs	1406	35.4	10.0	40.7	13.9	76.1	23.9	0.069	0.161
rRNAs	1931	50.4	7.7	28.2	13.7	78.6	21.4	-0.283	0.278
Control Region	1078	45.1	7.8	39.1	8.1	84.2	15.9	-0.072	0.018
Ricania fumosa									
Full genome	16016	28.0	14.7	48.1	9.3	76.1	24.0	0.264	-0.223
PCGs	10956	43.2	12.6	32.4	11.8	75.6	24.4	-0.142	-0.033

1st codon position	3652	36.8	11.3	35.5	16.4	72.3	27.7	-0.019	0.185
2nd codon position	3652	48.6	18.8	19.4	13.3	68.0	32.1	-0.430	-0.173
3rd codon position	3652	44.1	7.7	42.4	5.8	86.5	13.5	-0.020	-0.146
tRNAs	1411	35.8	10.6	40.3	13.3	76.1	23.9	0.060	0.110
rRNAs	1934	49.5	8.3	28.4	13.8	77.9	22.1	-0.271	0.249
Control Region	1652	33.2	14.3	42.4	10.0	75.6	24.3	0.121	-0.176

Table S8. Start and stop codons of eight Ricaniidae mitochondrial genomes.

Gene	Start codon/stop codon							
	Pochazia confusa	*Pochazia discreta*	*Pochazia guttifera*	*Ricania simulans*	*Ricania fumosa*	*Ricania speculum*	*Pochazia shantungensis*	*Ricania marginalis*
nad2	ATT/TAA	ATT/TAA	ATT/TAA	ATT/TAA	ATT/TAA	ATT/TAG	ATT/TAA	ATT/TAA
cox1	ATG/TAA	ATG/TAA	ATG/TAA	ATG/TAA	ATG/TAA	ATG/TAA	ATG/TAA	ATA/TAA
cox2	ATA/T	ATA/T	ATA/T	ATA/T	ATA/T	ATT/T	ATA/T	ATA/T
atp8	ATA/TAA	ATT/TAA	ATT/TAA	ATA/TAA	ATT/TAA	ATA/TAA	ATT/TAA	ATA/TAA
atp6	ATA/T	ATA/T	ATA/T	ATA/T	ATA/T	ATA/T	ATA/T	ATA/TAG
cox3	ATG/TAA	ATG/TAA	ATG/TAA	ATG/TAA	ATG/TAA	ATG/TAG	ATG/TAA	ATG/TAG
nad3	ATA/TAA	ATA/TAA	ATA/TAA	ATA/TAG	ATA/TAA	ATA/TAA	ATA/TAA	ATA/TAA
nad5	ATG/TAA	ATG/TAG	ATG/TAA	GTG/TAA	TTG/TAG	ATG/TAA	ATT/TAG	ATT/TAA
nad4	ATG/T	ATG/T	ATG/T	ATG/T	ATG/T	ATG/T	ATG/T	ATG/T
nad4L	ATG/TAA	ATG/TAA	ATG/TAA	ATT/TAA	ATG/TAA	ATG/TAA	ATG/TAA	ATG/TAA
nad6	ATA/TAA	ATA/TAA	ATA/TAA	ATC/TAA	ATA/TAA	ATC/TAA	ATA/TAA	ATC/TAA
cytb	ATG/TAA	ATG/TAA	ATG/TAA	ATG/TAA	ATG/TAA	ATG/TAA	ATG/TAA	ATG/TAA
nad1	ATG/T	ATG/T	ATG/T	ATG/T	ATG/T	ATG/A	ATG/A	ATG/TAA

Table S9. Best partitioning schemes and models based on different datasets for IQ-TREE analysis.

Dataset	Partitioning scheme	Best Models
PCG	*nad6*_codon1, *nad3*_codon1, *atp6*_codon1	GTR+I+G
	*cox1*_codon2, *cox3*_codon2, *atp6*_codon2, *cox2*_codon2, *cytb*_codon2	GTR+I+G
	*atp8*_codon3, *nad6*_codon3, *atp6*_codon3, *nad3*_codon3	TRN+G
	*atp8*_codon1, *nad2*_codon1	TVM+I+G
	*atp8*_codon2, *nad6*_codon2, *nad3*_codon2, *nad2*_codon2	TVM+I+G
	*cox1*_codon1	GTR+I+G
	*cox1*_codon3, *cytb*_codon3, *cox2*_codon3, *cox3*_codon3	K81UF+I+G
	*cytb*_codon1, *cox3*_codon1, *cox2*_codon1	GTR+I+G
	*nad1*_codon1, *nad4L*_codon1, *nad5*_codon1, *nad4*_codon1	GTR+I+G
	*nad4L*_codon2, *nad1*_codon2, *nad4*_codon2, *nad5*_codon2	GTR+I+G
	*nad1*_codon3	TRN+G
	*nad2*_codon3	K81UF+G
	*nad5*_codon3, *nad4L*_codon3, *nad4*_codon3	HKY+G
PCGR	*nad6*_codon1, *nad3*_codon1, *atp6*_codon1	GTR+I+G
	*cox3*_codon2, *atp6*_codon2, *cox1*_codon2, *cox2*_codon2, *cytb*_codon2	GTR+I+G
	*atp8*_codon3, *nad6*_codon3, *atp6*_codon3, *nad3*_codon3	TRN+G
	*atp8*_codon1, *nad2*_codon1	TVM+I+G
	*atp8*_codon2, *nad6*_codon2, *nad3*_codon2, *nad2*_codon2	TVM+I+G
	*cox1*_codon1	GTR+I+G
	*cox1*_codon3, *cytb*_codon3, *cox2*_codon3, *cox3*_codon3	K81UF+I+G
	*cytb*_codon1, *cox3*_codon1, *cox2*_codon1	GTR+I+G
	*nad1*_codon1, *nad4L*_codon1, *nad4*_codon1, *nad5*_codon1	GTR+I+G
	*nad4L*_codon2, *nad1*_codon2, *nad5*_codon2, *nad4*_codon2	GTR+I+G
	*nad1*_codon3	TRN+G
	*nad2*_codon3	K81UF+G
	*nad5*_codon3, *nad4L*_codon3, *nad4*_codon3	HKY+G
	rrnL, rrnS	GTR+I+G
PCG12	*atp6, nad3*	TIM+I+G
	atp8, nad2, nad6	GTR+I+G
	cox1	GTR+I+G
	cytb, cox2, cox3	GTR+I+G
	nad1	GTR+G
	nad4L, nad5, nad4	GTR+I+G
PCG12R	*atp6, nad3*	TIM+I+G
	atp8, nad6, nad2	GTR+I+G
	cox1	GTR+I+G
	cytb, cox3, cox2	GTR+I+G
	nad1, nad4L, nad4, nad5	GTR+I+G
	rrnL, rrnS	GTR+I+G
PCG-AA	*atp8, nad6, nad3, atp6, cox3, nad2*	MTART+I+G+F
	cox1, cox2, cytb	MTART+I+G+F
	nad1, nad4, nad4L, nad5	MTART+I+G+F

Table S10. Best partitioning schemes and models based on different datasets for MrBayes analysis.

Dataset	Partitioning scheme	Best Models
PCG	*nad3*_codon1, *atp6*_codon1, *cytb*_codon1, *cox3*_codon1, *cox2*_codon1	GTR+I+G
	*cox1*_codon2, *cox3*_codon2, *atp6*_codon2, *cox2*_codon2, *cytb*_codon2	GTR+I+G
	*atp8*_codon3, *nad6*_codon3, *nad3*_codon3, *atp6*_codon3	GTR+G
	*atp8*_codon1, *nad6*_codon1, *nad2*_codon1	GTR+I+G
	*atp8*_codon2, *nad2*_codon2, *nad3*_codon2, *nad6*_codon2	GTR+I+G
	*cox1*_codon1	GTR+I+G
	*cox1*_codon3, *cytb*_codon3, *cox3*_codon3, *cox2*_codon3	GTR+I+G
	*nad1*_codon1, *nad4*_codon1, *nad5*_codon1, *nad4L*_codon1	GTR+I+G
	*nad1*_codon2, *nad4L*_codon2, *nad5*_codon2, *nad4*_codon2	GTR+I+G
	*nad1*_codon3	GTR+G
	*nad2*_codon3	HKY+G
	*nad5*_codon3, *nad4L*_codon3, *nad4*_codon3	HKY+G
PCGR	*nad3*_codon1, *atp6*_codon1, *cytb*_codon1, *cox3*_codon1, *cox2*_codon1	GTR+I+G
	*cox1*_codon2, *cox3*_codon2, *atp6*_codon2, *cox2*_codon2, *cytb*_codon2	GTR+I+G
	*atp8*_codon3, *nad6*_codon3, *nad3*_codon3, *atp6*_codon3	GTR+G
	*atp8*_codon1, *nad6*_codon1, *nad2*_codon1	GTR+I+G
	*atp8*_codon2, *nad2*_codon2, *nad3*_codon2, *nad6*_codon2	GTR+I+G
	*cox1*_codon1	GTR+I+G
	*cox1*_codon3, *cytb*_codon3, *cox3*_codon3, *cox2*_codon3	GTR+I+G
	*nad1*_codon1, *nad4*_codon1, *nad5*_codon1, *nad4L*_codon1	GTR+I+G
	*nad1*_codon2, *nad4L*_codon2, *nad5*_codon2, *nad4*_codon2	GTR+I+G
	*nad1*_codon3	GTR+G
	*nad2*_codon3	HKY+G
	*nad5*_codon3, *nad4L*_codon3, *nad4*_codon3	HKY+G
	rrnL, rrnS	GTR+I+G
PCG12	*atp6, nad3*	GTR+I+G
	atp8, nad6, nad2	GTR+I+G
	cox1	GTR+I+G
	cytb, cox2, cox3	GTR+I+G
	nad1	GTR+G
	nad4L, nad5, nad4	GTR+I+G
PCG12R	*atp6, nad3*	GTR+I+G
	atp8, nad2, nad6	GTR+I+G
	cox1	GTR+I+G
	cytb, cox3, cox2	GTR+I+G
	nad1, nad4L, nad5, nad4	GTR+I+G
	rrnS, rrnL	GTR+I+G
PCG-AA	*cox2, cytb, atp6, cox3, atp8*	MTREV+I+G
	cox1	MTREV+I+G
	nad1, nad3	MTREV+G
	nad4L, nad2	MTREV+I+G
	nad4	MTREV+I+G

nad5	CPREV+I+G
nad6	MTREV+G
