# Supplementary figures and images for: Comparative Analysis of the Complete Mitochondrial Genomes of Five Species of Ricaniidae (Hemiptera: Fulgoromorpha) and Phylogenetic Implications
Source: Biology (Basel). 2022 Jan 7;11(1):92. doi: 10.3390/biology11010092 (PMC8772989; doi:10.3390/biology11010092)

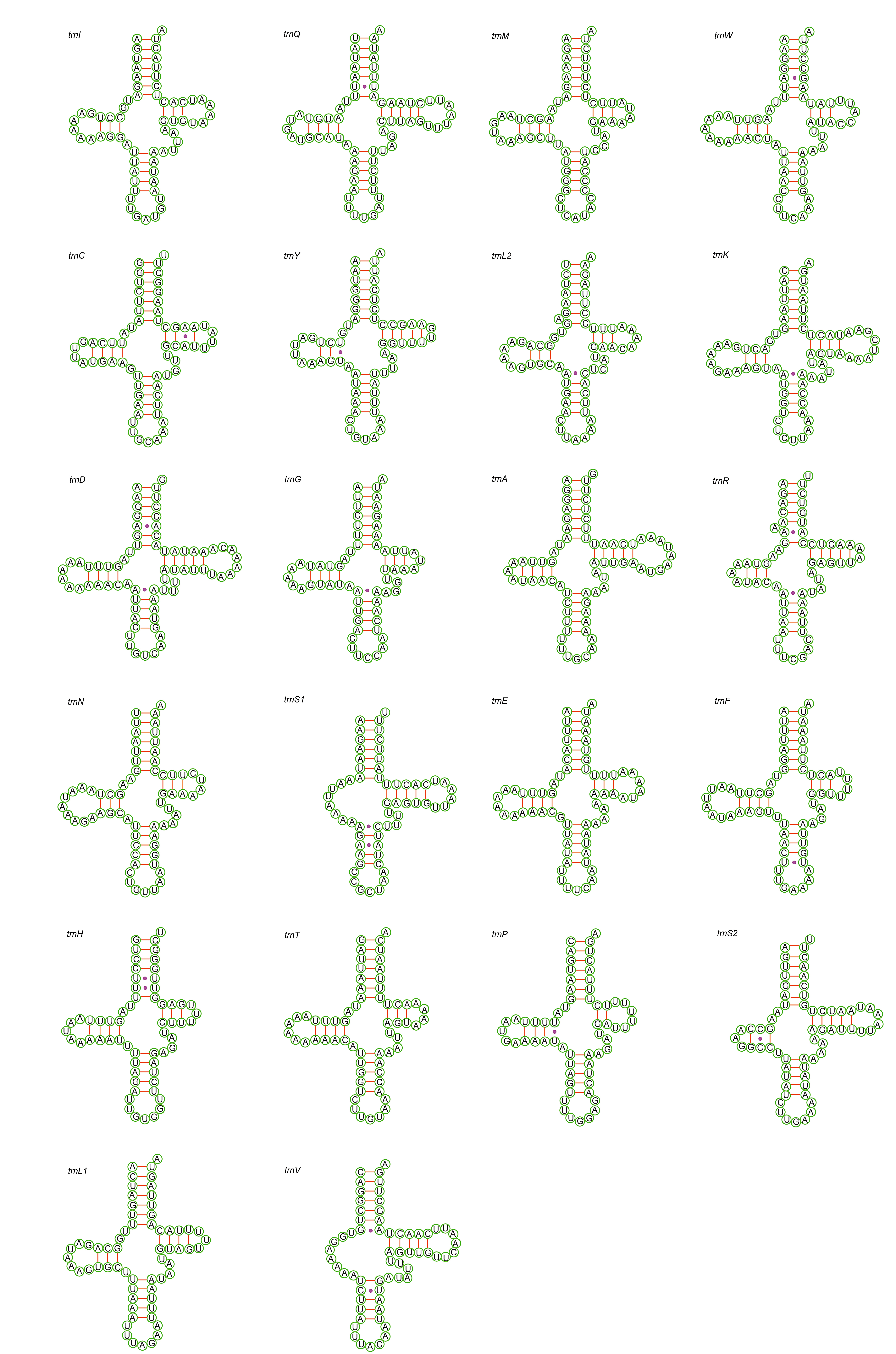

Supplement: Supplementary file 1 [file biology-11-00092-s001.zip › Figure S1 tRNA Pochazia confusa.jpg]

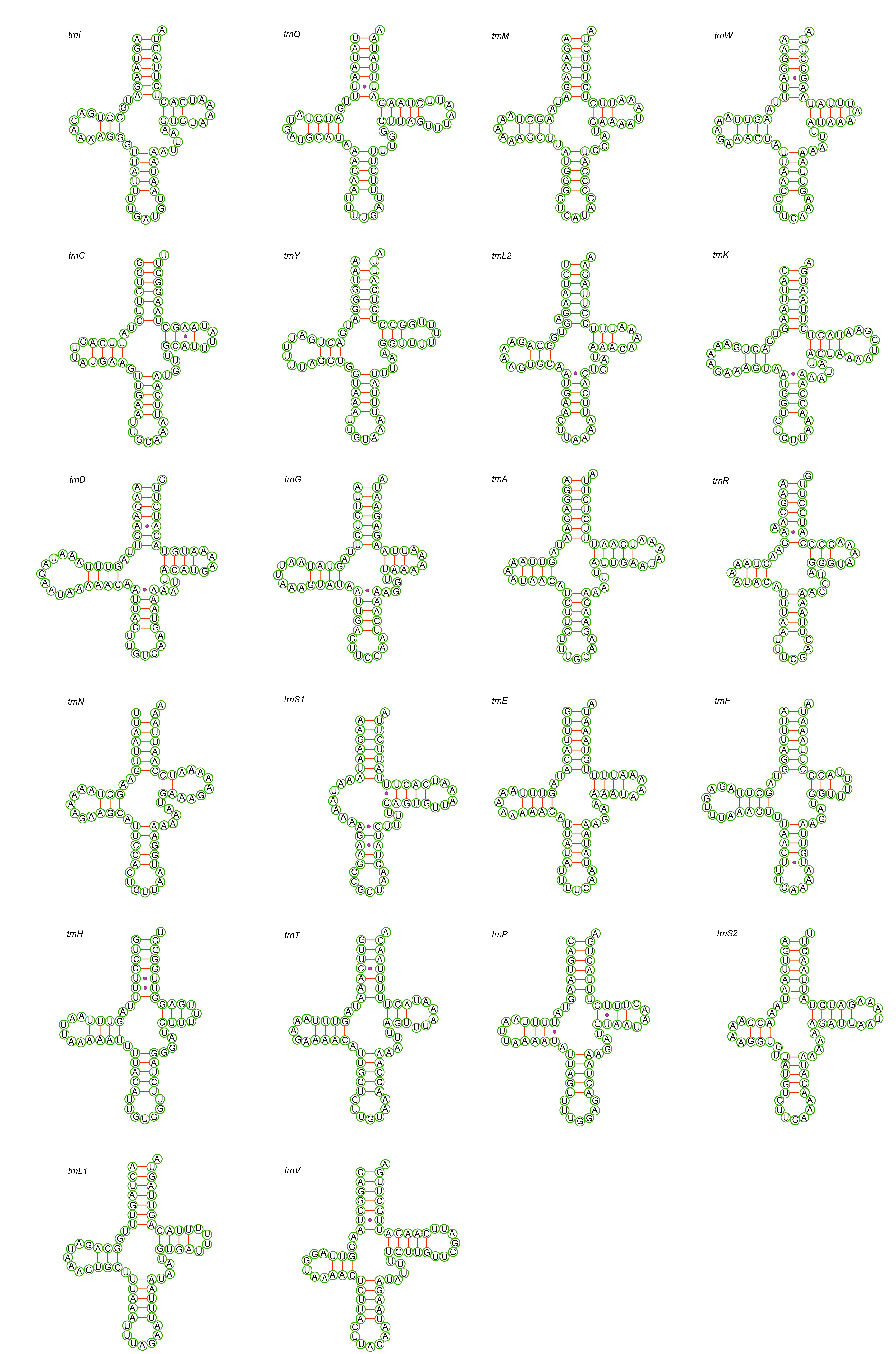

Supplement: Supplementary file 1 [file biology-11-00092-s001.zip › Figure S2 tRNA Pochazia discreta.jpg]

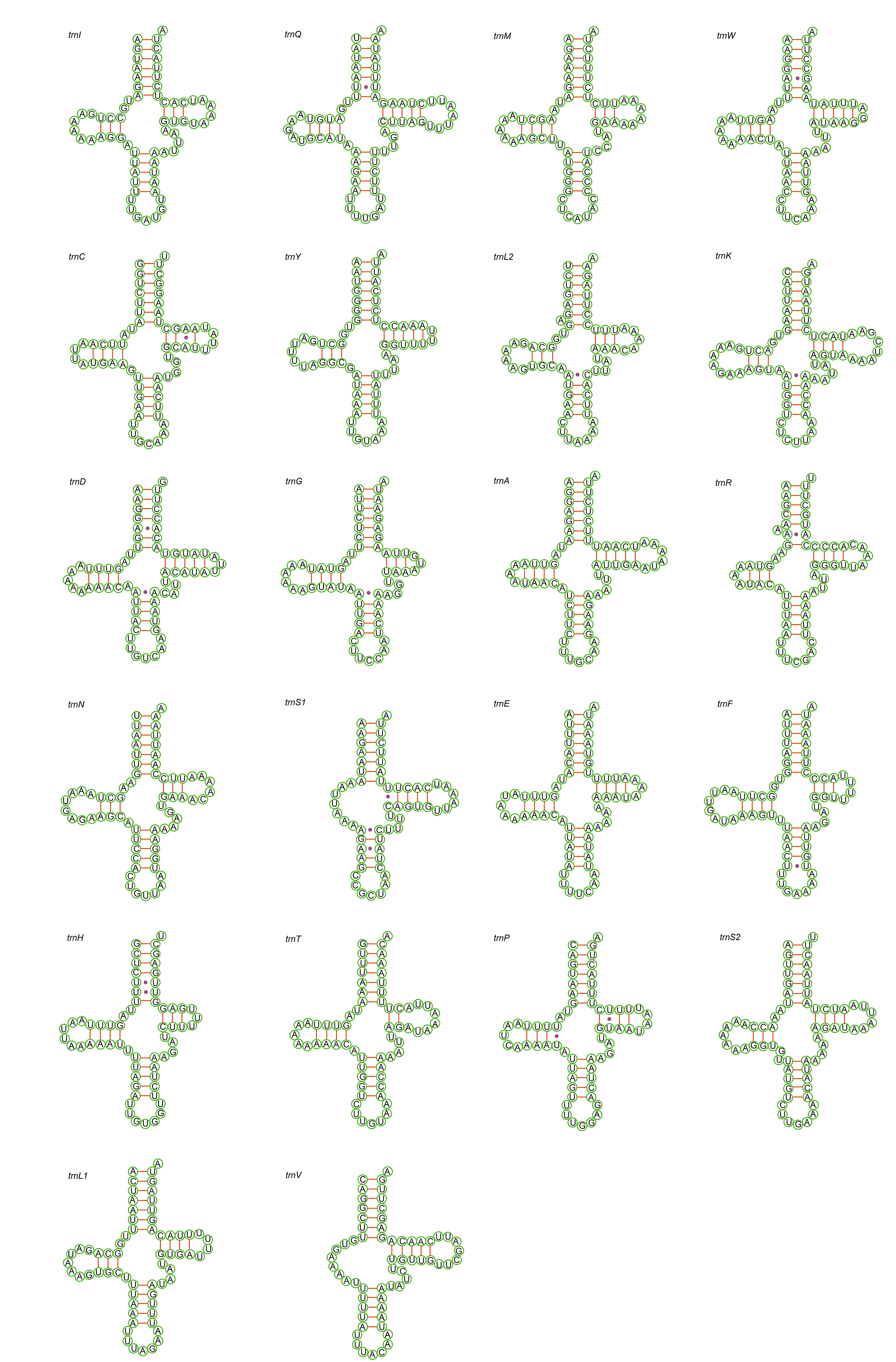

Supplement: Supplementary file 1 [file biology-11-00092-s001.zip › Figure S3 tRNA Pochazia guttifera.jpg]

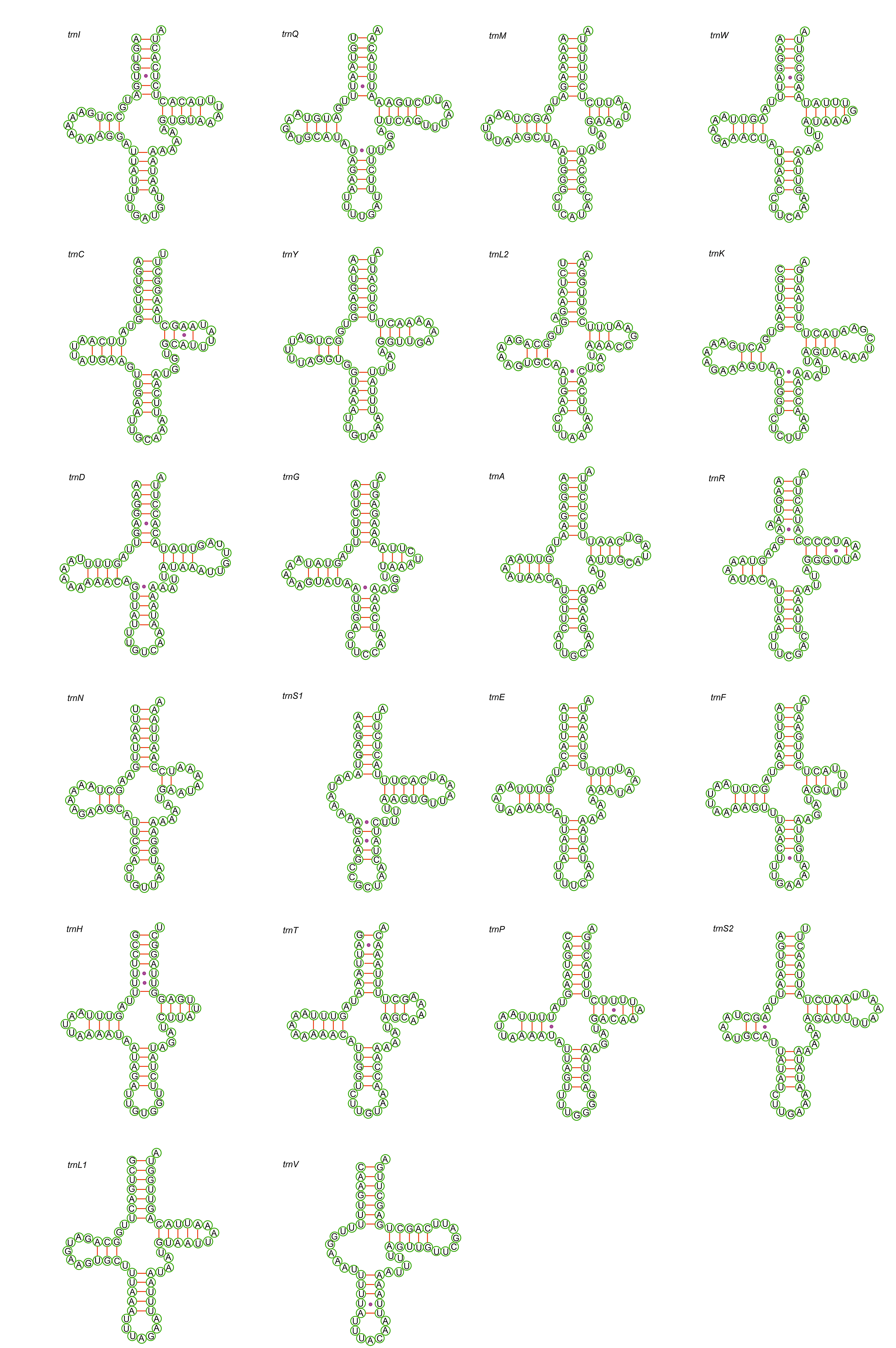

Supplement: Supplementary file 1 [file biology-11-00092-s001.zip › Figure S4 tRNA Ricania simulans.jpg]

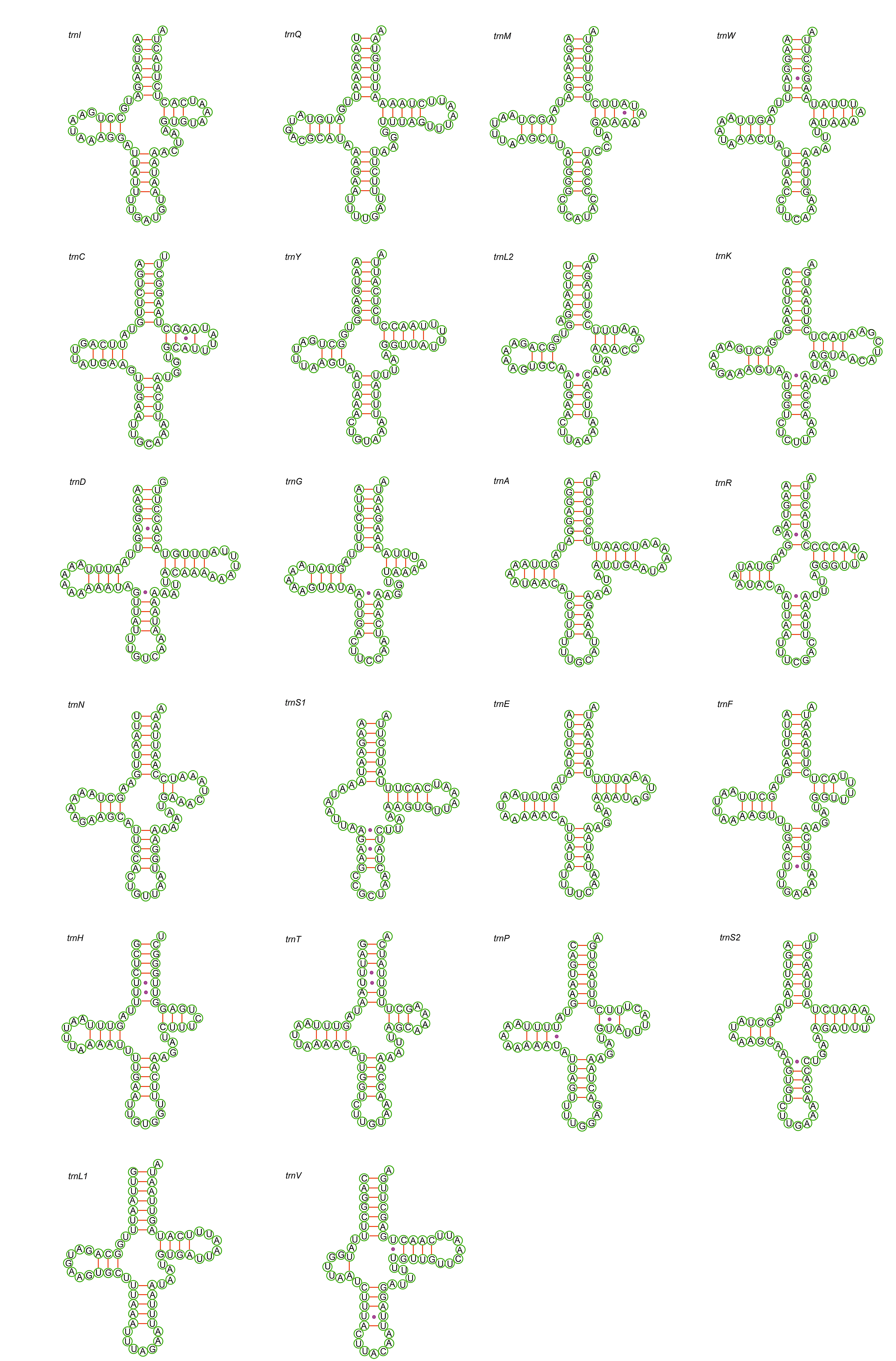

Supplement: Supplementary file 1 [file biology-11-00092-s001.zip › Figure S5 tRNA Ricania fumosa.jpg]

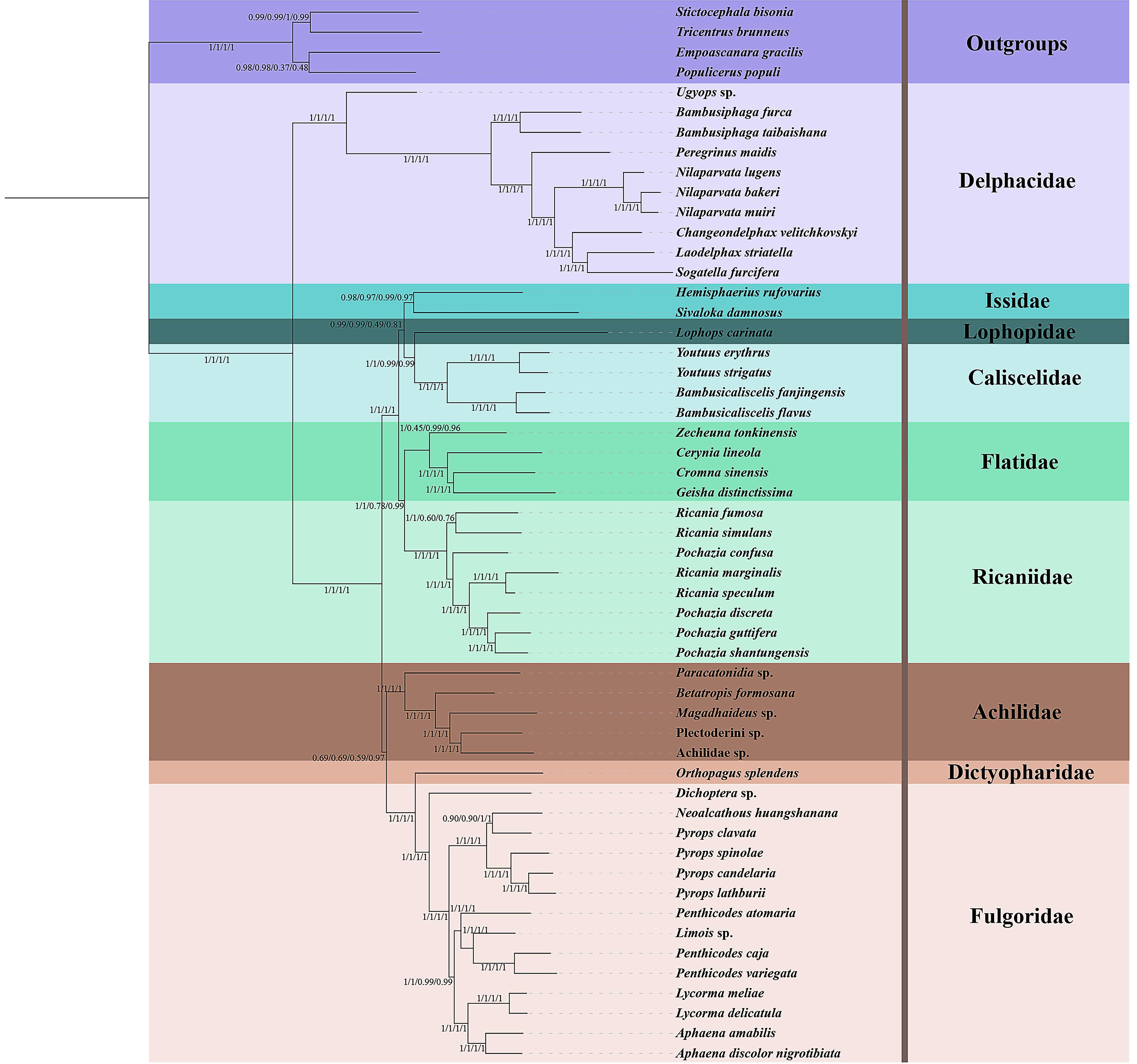

Supplement: Supplementary file 1 [file biology-11-00092-s001.zip › Figure S6. Phylogenetic trees obtained from PhyloBayes based on the data sets of PCG, PCGRNA, PCG12 and PCG12RNA.jpg]

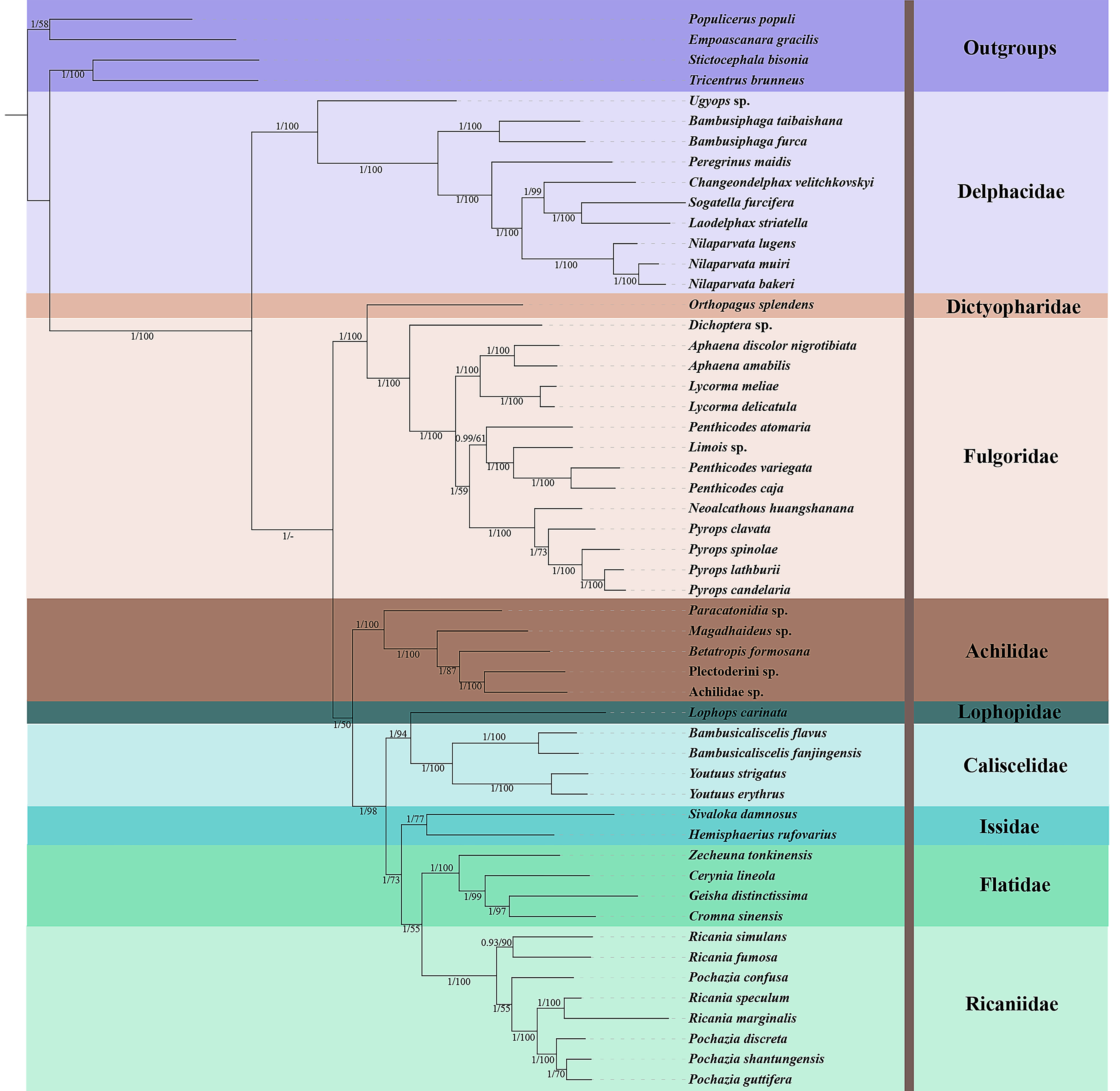

Supplement: Supplementary file 1 [file biology-11-00092-s001.zip › Figure S7. Phylogenetic trees obtained from IQ-TREE and MrBayes based on the data sets of PCG-AA.jpg]

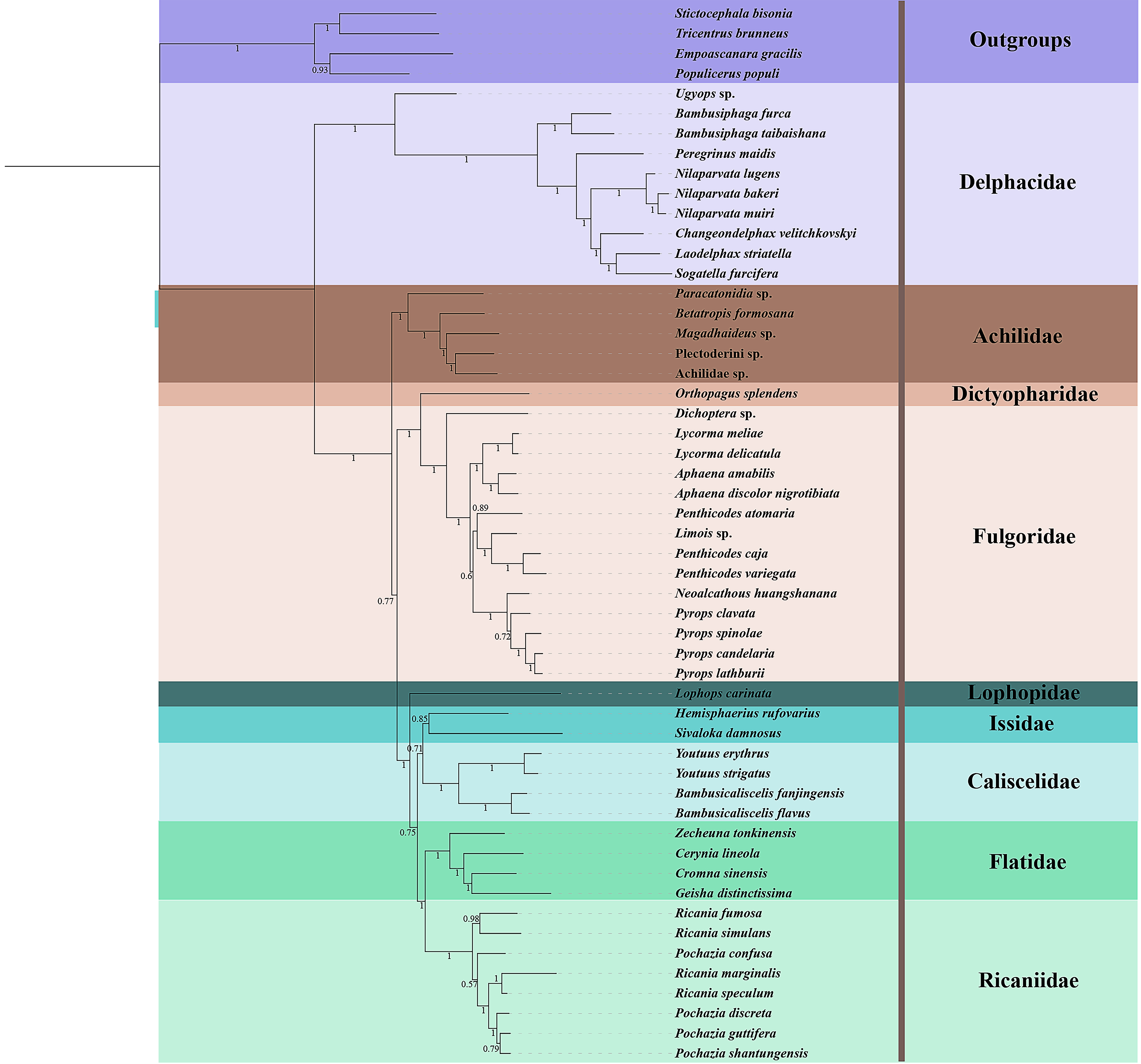

Supplement: Supplementary file 1 [file biology-11-00092-s001.zip › Figure S8. Phylogenetic trees obtained from PhyloBayes based on the data sets of PCG-AA.jpg]
